# N6‐methyladenosine regulatory machinery in plants: composition, function and evolution

**DOI:** 10.1111/pbi.13149

**Published:** 2019-05-21

**Authors:** Hong Yue, Xiaojun Nie, Zhaogui Yan, Song Weining

**Affiliations:** ^1^ College of Life Sciences State Key Laboratory of Crop Stress Biology in Arid Areas Northwest A&F University Yangling Shaanxi China; ^2^ College of Horticulture and Forestry Sciences Huazhong Agricultural University Wuhan Hubei China

**Keywords:** m6A, plant, epitranscriptome, RNA modification, regulatory machinery

## Abstract

N6‐methyladenosine (m6A) RNA methylation, one of the most pivotal internal modifications of RNA, is a conserved post‐transcriptional mechanism to enrich and regulate genetic information in eukaryotes. The scope and function of this modification in plants has been an intense focus of study, especially in model plant systems. The characterization of plant m6A writers, erasers and readers, as well as the elucidation of their functions, is currently one of the most fascinating hotspots in plant biology research. The functional analysis of m6A in plants will be booming in the foreseeable future, which could contribute to crop genetic improvement through epitranscriptome manipulation. In this review, we systematically analysed and summarized recent advances in the understanding of the structure and composition of plant m6A regulatory machinery, and the biological functions of m6A in plant growth, development and stress response. Finally, our analysis showed that the evolutionary relationships between m6A modification components were highly conserved across the plant kingdom.

## Introduction

RNA molecules are essential components in all living organisms. These molecules act as carriers to pass genetic information from DNA to protein and as regulators of all kinds of biological processes (Fu *et al*., 2014). RNA transcripts may undergo diverse and complex chemical modifications to tailor their structure to a particular molecular function. Indeed, more than one hundred types of post‐transcriptional modifications have been identified in cellular RNA. These include modifications at the nascent pre‐mRNA stage, even before the splicing process occurs. The importance of mRNA modifications in epigenetics has been neglected over the past several decades due to the low abundance of mRNA chemical modifications and limitations in research methods to detect such modifications. Among the post‐transcriptional modifications, methylation of adenosine in the N6 position (m6A) is the most prevalent internal modification and extensively present in rRNAs, mRNA, tRNAs, miRNA and long non‐coding RNA (Cantara *et al*., 2010; Wei *et al*., 1975, 2017). It is noteworthy that, in eukaryotic cells, m6A accounts for up to 80% of all RNA methylation modifications and 50% of methylated nucleotides in polyadenylated mRNA (Kierzek and Kierzek, 2003). m6A was first discovered in wheat (*Triticum turgidum* L.), oat (*Avena sativa* L.) coleoptiles and maize (*Zea mays* L.) approximately 40 years ago and subsequently widely identified in viruses, flies, yeast, plants, human and other mammals (Haugland and Cline, 1980; Jia *et al*., 2013; Kennedy and Lane, 1979; Nichols and Welder, 1981). In previous studies, the m6A modification was believed to be ‘static’. However, after the discovery of the first m6A RNA demethylase, fat mass and obesity‐associated protein (FTO), it became evident that RNA modifications could be dynamic and reversible (Jia *et al*., 2011). Subsequently, the idea of the ‘epitranscriptome’ was proposed and gradually branched into a new research field (Hussain *et al*., 2013; Saletore *et al*., 2012). Since then, it has become increasingly clear that m6A is indispensable for the regulation of gene expression in living cells.

In mammals, there is a reported m6A modification rate of 0.1–0.4%, which is equivalent to an average of one m6A site per 2,000 ribonucleotides. A slightly higher rate of 0.7–0.9% is reported in the meiotic yeast *Saccharomyces cerevisiae* (Bodi *et al*., 2010; Fu *et al*., 2014; Wei *et al*., 1975). Between 1 and 15 m6A sites per RNA molecule have been suggested in various viruses, while *Arabidopsis thaliana* contains 0.5–0.7 sites per 1000 nucleotides or 0.7–1.0 sites per actively expressed transcript (Luo *et al*., 2014; Zhao *et al*., 2017; Zhong *et al*., 2008).

In plants and other eukaryotes, m6A is generated by the binding of m6A methyltransferase to a highly conserved consensus sequence, RRACH (R = G or A; H: U>A>C) (Shen *et al*., 2016). Interestingly, when the highly conserved GAC was mutated to GAU, m6A was no longer methylated in Rous sarcoma virus mRNA (Kane and Beemon, 1987). The frequency of m6A modification is not evenly distributed within RNA, being particularly highly enriched in mature mRNAs. This modification is always clustered in the stop codons and 3′untranslated regions (UTRs), especially at the 3′‐end of the coding sequence (CDS) and the first quarter of the 3′‐UTR (Dominissini *et al*., 2012, 2013; Ke *et al*., 2015; Meyer *et al*., 2012; Schwartz *et al*., 2013, 2014). In plants, m6A is similarly enriched in these regions, but also present in the start codon (Slobodin *et al*., 2017). More than 60% of m6A modifications are located in the start codon of chloroplast‐associated proteins and certain photosynthesis‐related genes also show an abundance of m6A sites, indicating that m6A may have unique functions associated with photosynthesis (Li *et al*., 2014b; Luo *et al*., 2014). Studies mapping m6A report that this modification is also strongly enriched in the 5′ UTR of human and other mammals under various stress conditions (Lee *et al*., 2015; Meyer and Jaffrey, 2017; Wang *et al*., 2015b; Zhou *et al*., 2015).

Until now, most m6A studies focus on human and other mammalian systems, while little research has been devoted to exploring m6A in plants at the molecular level. Additionally, many previous theories on m6A modifications in human and other mammals have been challenged in plant systems. In this review, we firstly identified m6A readers, writers and erasers in 22 plant species to conclude the composition and structure of m6A machinery in plants. Due to the high conservation of m6A structural machinery in different species, we selected *Arabidopsis* as a model to determine the homologous components in other plant species, with the aim of revealing shared underlying molecular mechanisms of m6A modification. Secondly, we systematically reviewed the recent advances in the understanding of the biological functions of m6A methylation in plants. Thirdly, we explored the evolution of relationships between m6A methylation compositions across the plant kingdom. The composition, function and evolution of m6A in plants reviewed in this study will contribute to better understand the functions of m6A, and also help to reveal the complexity of RNA modification regulatory mechanisms.

## The composition of the m6A regulatory network: writers, erasers and readers

**Figure 1:**
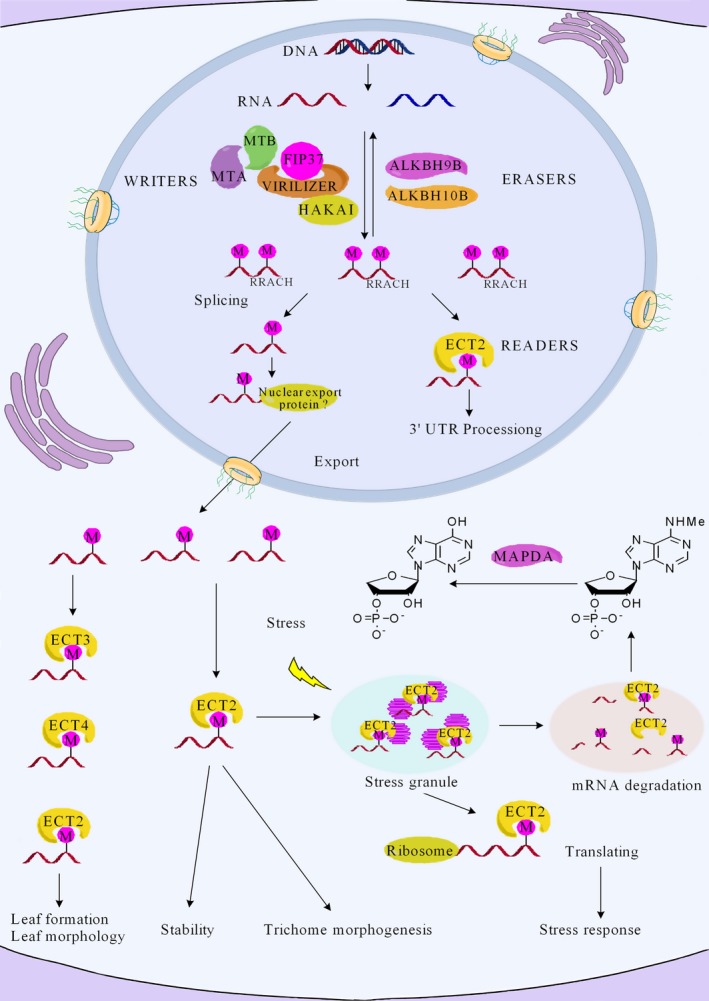
A working model for the regulation of mRNA stability and translation in *Arabidopsis*, through the action of a network of m6A writer, eraser and reader proteins. m6A methyltransferases (writers) and demethylases (erasers) lead to the dynamic patterning of m6A modifications in mRNA. The m6A writer complex includes the proteins MTA, MTB, FIP37, VIRILIZER and HAKAI. The m6A modifications can be removed by ALKBH9B and ALKBH10B proteins within nucleus. The ECT2/3/4 and CPSF30 proteins serve as m6A readers, which bind specifically to m6A sites (RRACH) and mediate specific functions. The vital role of m6A methylation in mRNA metabolism, translation and stability has been uncovered. The protein ECT2 regulates 3′ UTR mRNA processing in the nucleus. However, after ECT2 is exported to the cytoplasm it can bind to m6A‐containing RNAs to promote mRNA translation and direct mRNAs to stress granules for improved stress tolerance. The other reader proteins ECT3/4 may regulate leaf formation and morphology in *Arabidopsis*. Finally, m6A modifications in RNA may be converted to N6‐mAMP, with the enzyme MAPDA catalysing N6‐mAMP to IMP.

The m6A regulatory machinery is post‐transcriptionally assembled by a conserved set of proteins at the conserved consensus sequence, RRACH. A number of proteins involved in the addition, removal and identification of m6A have been reported, which are categorized into three groups called writers, erasers and readers, respectively, and include proteins such as MTA, MTB, ALKBH9B and ECT2 (Figure 1) (Arribas‐Hernández *et al*., 2018; Fu *et al*., 2014; Martínez‐Pérez *et al*., 2017; Zhong *et al*., 2008). The m6A writer complex recognizes the consensus motif RRACH (Růžička *et al*., 2017). However, not all RRACH motifs are associated with m6A modification, as the m6A level is much lower than the abundance of RRACH motifs, indicating that the molecular mechanism regulating m6A modification is not fully understood (Liu and Pan, 2016). The *Arabidopsis thaliana* proteins ALKBH9B and ALKBH10B function as m6A erasers (RNA demethylases) that oxidatively reverse m6A methylation from single‐stranded RNA molecules (Duan *et al*., 2017; Martínez‐Pérez *et al*., 2017). YTH domain proteins act as m6A readers. For instance, ECT2 is one of the most important readers, which is significantly enriched in the 3′UTRs of target genes. It plays a vital role in the regulation of 3′ UTR processing in the nucleus and in controlling RNA stability in the cytoplasm. When RNA molecules were exported from the nucleus to the cytoplasm, ECT2 combined with transcripts that are associated with trichome morphogenesis to controlling trichome branching. ECT3 and ECT4 can bind to a specific site on m6A‐modified cellular RNAs in the cytoplasm. ECT2/3/4 proteins are required in the timing and regulation of leaf formation, and normal leaf morphology. In addition, during the inhibition of translation initiation in *Arabidopsis* under heat stress, ECT2 relocates to stress granules, suggesting that it may also control mRNA fate in the cytosol (Arribas‐Hernández *et al*., 2018; Scutenaire *et al*., 2018; Wei *et al*., 2018). Finally, the turnover of RNA *in vivo* generates N6‐methylated AMP (N6‐mAMP), which is converted by the enzyme MAPDA to inosine monophosphate (IMP) through a hydrolytic reaction (Figure 1) (Chen *et al*., 2018; Lockhart, 2018a). We postulate that a better understanding of the molecular mechanisms underlying m6A modification in *Arabidopsis* will provide further insights into m6A processing in plants, as well as other model systems.

## m6A writers

The first m6A methyltransferase identified in mammals was named METTL3 and was cloned from the 200 kDa methylase complex (Bokar *et al*., 1997). It is one member of the putative S‐adenosyl‐L‐methionine (SAM)‐dependent methyltransferase family that is highly conserved in plants and mammals (Meyer and Jaffrey, 2017). Extensive research on m6A writers has been conducted in flies, human and other mammals over the past few years, with vital m6A methyltransferase components also being characterized in *Arabidopsis*. MTA (METTL3 human homolog protein) is one of the earliest discovered methyltransferases in *Arabidopsis*. Subsequently, evolutionary analysis and experimental investigation suggested that the METTL14 protein was the second most‐active m6A methyltransferase enzyme in human to catalyse m6A RNA methylation, being highly homologous to METTL3 (Bujnicki *et al*., 2002; Liu *et al*., 2014). However, this theory was overturned two years later when studies found that METTL14 did not have methyltransferase activity. Rather, METTL14 had a primary role in binding to RNA substrates in mammalian cells, before enabling their interaction with METTL3 through a hydrogen bonding network to form a very stable anti‐parallel heterodimer (Śledź and Jinek, 2016; Wang *et al*., 2016a,b). MTB (METTL14 human homolog protein) has been identified in *Arabidopsis,* but its function remains unknown (Arribas‐Hernández *et al*., 2018). Depletion of the pre‐mRNA splicing regulator, WTAP, can also lead to a significant decrease in m6A, indicating that it is the third major binding partner of the methylation complex. WTAP plays a vital role in initiating and controlling the localization of foci within the nucleus that are enriched with pre‐mRNA splicing factors required for activity of the METTL3‐METTL14 complex (Liu *et al*., 2014; Ping *et al*., 2014; Schwartz *et al*., 2014). FIP37 (WTAP human homolog protein), an E3 ubiquitin ligase, was first identified in *Arabidopsis* to interact with MTA. The fourth key component of the m6A methylation complex, KIAA1429, was identified through knockout mutations to cause substantial loss of m6A in mammals (Schwartz *et al*., 2014). Virilizer is the homologous protein in fly, which catalyses m6A formation to control sex determination, and is considered the fifth component of m6A writers (Hilfiker *et al*., 1995; Kan *et al*., 2017). In *Arabidopsis*, VIRILIZER (KIAA1429 human homolog protein) and the E3 ubiquitin ligase HAKAI (HAKAI human homolog protein) have been found as the fourth and fifth key component, respectively (Bodi *et al*., 2012; Růžička *et al*., 2017).

m6A writers are highly conserved. Thus, the protein components in *Arabidopsis* can provide the sequence information required to identify the orthologous genes in different plant species. As much plant genome information is publically available, a number of representative species were selected on which to perform comparative genomics analysis, including six dicotyledonous species, six monocotyledon species, one pteridophyte species, two moss species and seven algae species (detailed information is listed in Table 1). The protein sequences for these species were obtained from the Ensembl plants database (http://plants.ensembl.org/index.html) and National Center for Biotechnology Information (NCBI). Local protein databases were constructed from these downloaded protein sequences and subsequently used to search for candidate m6A writer components, with the known *Arabidopsis* proteins atMTA, atMTB, atFIP37, atVIRILIZER and atHAKAI used as a reference. The HMM (hidden Markov model) profile for the MTA70 superfamily (PF05063), WTAP superfamily (PF17098) and virilizer motif (PF15912) sequences was download from the PFAM database, and the HMMER search tool was used to assess all plant species protein sequences (Finn *et al*., 2006; Wheeler and Eddy, 2013). As shown in Table 1, 159 putative m6A writer components were identified using profile HMM searches and BLASTP, and the sequence information is shown in Table S1. A total of 69 MTA, MTB and MTC proteins were identified from all plants. AtMTA proteins are mainly distributed in dividing tissues, especially there production organs, apical meristems and newborn root. Inactivation of AtMTA proteins prevents development, arresting plant embryos at the globular stage, and eventually leads to embryo lethality. m6A modification reduces the relative abundance of MTA proteins in reproduction organs, shoot and lateral roots meristems (Zhong *et al*., 2008). Interestingly, only one MTA was discovered in barley and *Micromonas pusilla*. In these two species, the MTB and MTC proteins may have adopted the specific molecular role of the MTA protein. There is also limited functional knowledge on the AtMTC protein, another m6A writer component identified in this review.

**Table 1:** Characteristics of the putative m6A writers, erasers and readers in 22 plant species

Species	m6A Writers				m6A Erasers		m6A Readers			
*Arabidopsis thaliana*	AtMTA	AtFIP37	AtVIR	AtHAKAI	AtALKBH9B	AtALKBH10B	AtECT2	AtECT3	AtECT4	AtCPSF30
	AtMTB									
	AtMTC									
*Cicer arietinum*	CaMTA	CaFIP37‐1	CaVIR1	CaHAKAI	CaALKBH3B	CaALKBH16B	AtECT2	AtECT3	AtECT4	AtCPSF30
	CaMTB	CaFIP37‐2	CaVIR2			CaALKBH17B	CaECT8	CaECT6	‐	CaECT19
	CaMTC	CaFIP37‐3	CaVIR3							
		CaFIP37‐4								
*Vitisvinifera*	VvMTA	VvFIP37	VvVIR	VvHAKAI	VvALKBH6B	VvALKBH10B	VvECT9	VvECT1	VvECT11	VvECT3
	VvMTB									
	VvMTC									
*Solanum lycopersicum*	SlMTA	SlFIP37	SlVIR	SlHAKAI	SlALKBH3B	SlALKBH10B	SlECT1	SlECT2	‐	SlECT4
	SlMTB				SlALKBH4B			SlECT9		SlECT6
	SlMTC									
	SlMTD									
*Brassica rapa*	BrMTA	BrFIP37‐1	BrVIR	BrHAKAI	BrALKBH6B	BrALKBH12B	BrECT16	BrECT1	BrECT14	BrCPSF30‐1
	BrMTB	BrFIP37‐2						BrECT7	BrECT15	BrCPSF30‐2
	BrMTC									
*Gossypiumhirsutum*	GhMTA	GhFIP37‐1	GhVIR1	GhHAKAI1	GhALKBH9B	GhALKBH11B	GhECT15	GhECT5	GhECT18	GhECT20
	GhMTB	GhFIP37‐2	GhVIR2	GhHAKAI2	GhALKBH10B	GhALKBH23B	GhECT16	GhECT6		GhECT27
	GhMTC		GhVIR3	GhHAKAI3						
	GhMTD			GhHAKAI4						
	GhMTE			GhHAKAI5						
*Chenopodium quinoa*	CqMTA				CqALKBH13B	CqALKBH26B	CqECT14	CqECT15	CqECT9	CqCPSF30‐1
	CqMTB	CqFIP37‐1	CqVIR1	CqHAKAI1		CqALKBH27B				CqCPSF30‐2
	CqMTC	CqFIP37‐2	CqVIR2	CqHAKAI2						
	CqMTD									
	CqMTE									
	CqMTF									
*Zea mays*	ZmMTA	ZmFIP37‐1	ZmVIR1	ZmHAKAI1	ZmALKBH1B	ZmALKBH5B	ZmECT7	ZmECT8	ZmECT11	ZmCPSF30‐1
	ZmMTB	ZmFIP37‐2	ZmVIR2	ZmHAKAI2		ZmALKBH10B	ZmECT23		ZmECT25	
	ZmMTC	ZmFIP37‐3								
		ZmFIP37‐4								
		ZmFIP37‐5								
*Triticumaestivum*	TaMTA‐D	TaFIP37‐1D	TaVIR‐D	TaHAKAI1‐D	TaALKBH4B	TaALKBH6B	TaECT7	TaECT6	TaECT3	TaCPSF30‐2
	TaMTA‐A	TaFIP37‐2D	TaVIR‐A	TaHAKAI1‐A		TaALKBH29B	TaECT21	TaECT22	TaCPSF30‐4	TaCPSF30‐5
	TaMTC‐D	TaFIP37‐2A	TaVIR‐B	TaHAKAI1‐B						
	TaMTB‐A	TaFIP37‐1B		TaHAKAI2‐D						
	TaMTB‐D									
	TaMTA‐B									
	TaMTC‐B									
	TaMTB‐B									
	TaMTC‐A									
*Sorghum bicolor*	SbMTA	SbFIP37	SbVIR1	SbHAKAI1	SbALKBH13B	SbALKBH4B	SbCPSF30‐3	SbCPSF30‐1	‐	SbCPSF30‐19
	SbMTB		SbVIR2	SbHAKAI2	SbALKBH14B	SbALKBH15B	SbCPSF30‐4	SbCPSF30‐2		
	SbMTC						SbCPSF30‐8			
*Oryzaindica*	OiMTA	OiFIP37	OiVIR	OiHAKAI						
	OiALKBH6B	OiALKBH5B	OiECT10	OiECT7	‐	OiECT2				
	OiMTB					OiALKBH12B				
	OiMTC									
*Hordeum vulgare*	HvMTA	HvFIP37	HvVIR	HvHAKAI	HvALKBH1B	HvALKBH4B	HvECT2	HvECT4	HvECT1	‐
						HvALKBH8B	HvECT6		HvECT8	
*Selaginellamoellendorffi*	SmMTA	SmFIP37	–	–	SmALKBH4B	SmALKBH14B	‐	SmECT2	‐	SmECT1
	SmMTB					SmALKBH16B		SmECT4		SmECT3
*Marchantiapolymorpha*	MapMTA	MapFIP37	MapVIR1	MpHAKAI	MapALKBH6B	MapALKBH10B	‐	MapECT1	‐	MapECT3
	MapMTB		MapVIR2					MapECT2		
	MapMTC									
*Physcomitrella patens*	PpMTA	PpFIP37‐1	PpVIR	PpHAKAI1	PpALKBH5B	PpALKBH2B	‐	PpECT1	‐	PpECT4
	PpMTB	PpFIP37‐2		PpHAKAI2	PpALKBH6B	PpALKBH4B		PpECT3		
	PpMTC			PpHAKAI3						
	PpMTD									
*Cyanidioschyzonmerolae*	CmMTA	–	–	–	‐	CmALKBH1B	‐	‐	‐	‐
	CmMTB									
*Micromonaspusilla*	MipMTA	MipFIP37	–	–	‐	MipALKBH4B	MipECT1	‐	‐	MipCPSF30‐1
										MipCPSF30‐2
*Emilianiahuxleyi*	EhMTA	EhFIP37‐1	–	–	EhALKBH21B	EhALKBH18B	EhECT1	‐	‐	‐
	EhMTB	EhFIP37‐2				EhALKBH24B				
	EhMTC									
	EhMTD									
*Volvoxcarteri*	VcMTA	VcFIP37	–	–	VcALKBH1B	‐	‐	VcCPSF30‐1	‐	VcECT1
	VcMTB				VcALKBH4B					
	VcMTC									
*Ectocarpus siliculosus*	–	EsFIP37	–	–	EsALKBH2B	EsALKBH4B	‐	EsECT1	‐	EsECT2
						EsALKBH6B				EsCPSF30‐2
*Chlorella variabilis*	CvMTA	CvFIP37	CvVIR	CvHAKAI	CvALKBH2B	‐	‐	CvCPSF30‐1	‐	CvECT1
	CvMTB									CvCPSF30‐3
	CvMTC									
*Chlamydomonasreinhardtii*	CrMTA	CrFIP37‐1	CrVIR	–	CrALKBH1B	CrALKBH2B	‐	CrECT1	‐	CrECT2
	CrMTB	CrFIP37‐2								
	CrMTC	CrFIP37‐3								
	CrMTD									

Five ZmFIP37 and two TaFIP37 proteins were identified in maize and wheat, respectively. Delayed endosperm and embryo development, and subsequent embryonic lethality were observed on knockout of fip37 in *Arabidopsis* (Vespa *et al*., 2004; Zhong *et al*., 2008). Deletion of FIP37 was found to significantly reduce the m6A modifications within the 3′UTR and stop codons with less effect on m6A modifications within the 5′UTR. The function of FIP37 was found to be distinct from WTAP in animals. WTAP localizes to nuclear foci and affects the splicing of mRNA, while FIP37 is evenly distributed within the nucleoplasm and it is not found to affect RNA splicing (Bodi *et al*., 2012; Shen *et al*., 2016; Zhong *et al*., 2008). The distribution of FIP37 is similar to MAT, in that both proteins are highly expressed in apical meristems, young leaves and floral organs. FIP37 knockout plants display cellular over‐proliferation in shoot apical meristems, suggesting that m6A modification is essential for regulating cell division in the meristem (Shen *et al*., 2016). Consequently, we speculate that these FIP37s play an indispensable role for maintaining appropriate proliferation of the shoot meristem in plants (Yuan, 2017).

## m6A erasers

In mammals, the first identified m6A demethylase, FTO (fat mass and obesity‐associated protein), was found to catalyse the reversion of m6A to adenosine in a α‐KG (α‐ketoglutarate) and Fe^2+^‐dependent manner, suggesting that m6A is a reversible and dynamic modification. However, recent research has reported FTO as the eraser for m6Am (N6,20‐O dimethyladenosine), not m6A itself. Mauer *et al*. found that the catalytic rate of FTO was significantly increased when the substrate was m6Am compared with m6A (Mauer *et al*., 2017). In addition, FTO showed a strong preference for m6Am in consensus site analysis while it did not have a preference for m6A (Jia *et al*., 2011; Meyer and Jaffrey, 2017). A second m6A demethylase, ALKBH5 (alkylation repair homolog 5), is a homolog of FTO (Jia *et al*., 2013). A high abundance of ALKBH5 in breast cancer cells correlates with a decrease in the relative abundance of m6A (Zhang *et al*., 2016). ALKBH5 has catalytic activity when the substrate is m6A and no catalytic activity for m6A, suggesting that ALKBH5 is a mRNA m6A demethylase (Mauer *et al*., 2017).

Transcriptome‐wide profiling has revealed that m6A modification is also a dynamic process in *Arabidopsis* (Luo *et al*., 2014). Several studies have confirmed that m6A can revert to adenosine through the action of the m6A RNA demethylases ALKBH9B and ALKBH10B (Duan *et al*., 2017; Martínez‐Pérez *et al*., 2017). AtALKBH9B localizes to cytoplasmic granules, which contain siRNA bodies and can be directed to P bodies. ALKBH9B and ALKBH10B belong to the AlkB family of Fe (II)/α‐ketoglutarate‐dependent dioxygenases, containing a highly conserved clavaminate synthase‐like domain. The HMM profile of the clavaminate synthase‐like domain (PF13532) sequences was download, and the HMMER search tool was used to identify orthologous genes in 22 plant species. In total, 293 homologs of the *Escherichia coli* AlkB family were been identified in 22 plant species (Table S2). These studies have uncovered the processing of m6A demethylation in *Arabidopsis*, although the existence and function of demethylases in other plant species remain unclear.

## m6A Readers

To understand the molecular mechanism underlying m6A regulation of gene expression, it is vital to elucidate how m6A readers function. These reader proteins bind specifically to m6A‐modified cellular RNAs to implement the biological function of methylation modifications. The two m6A readers, YTHDF2 and YTHDF3, were discovered by RNA pulldown (Dominissini *et al*., 2012). Further studies found that m6A readers contained either a YTH (YT512‐B Homology) domain or eIF3 (eukaryotic initiation factor 3) (Wang *et al*., 2014, 2015b; Xiao *et al*., 2016). YTH family members are highly conserved and contain a YTH domain with an aromatic cage for m6A recognition. These proteins are widely found in humans, Drosophila, yeast and *Arabidopsis* (Li *et al*., 2014a; Meyer and Jaffrey, 2017). Additionally, combining different m6A reader proteins can result in distinct functions. For example, YTHDF1 is usually localized to the cytoplasm but may interact with eIF3 in the nucleus to promote translation initiation and protein synthesis (Wang *et al*., 2015b). The cytoplasmic protein YTHDF2 has more than 3000 target RNAs containing m6A, which can specifically recognize the conserved core motif G(m6A)C of m6A in most mRNA and some non‐coding RNAs. Interestingly, YTHDF1 and YTHDF2 share common target mRNAs. YTHDF1 promotes the efficient translation of target mRNAs, whereas YTHDF2 recognizes these target transcripts and facilitates their decay (Wang *et al*., 2015b). Furthermore, the m6A nuclear binding protein YTHDC1 mediates mRNA splicing (Xiao *et al*., 2016; Xu *et al*., 2015). YTHDC1 interacts with the precursor mRNA splicing factor SRSF3 to promote m6A binding and inhibits the binding between the splicing factor SRSF10 and m6A, leading to the reversal of m6A modifications (Xiao *et al*., 2016; Zhao *et al*., 2014). LncRNA XIST (X‐inactive specific transcript) can induce m6A methylation on XIST by binding the proteins RBM15 and RBM15B required to recruit methylase complexes. YTHDC1 binds to m6A sites to promote XIST‐mediated transcript silencing in the X chromosome gene (Patil *et al*., 2016). Consequently, the discovery of this phenomenon stimulated much research effort to determine the function of m6A readers.

**Figure 2:**
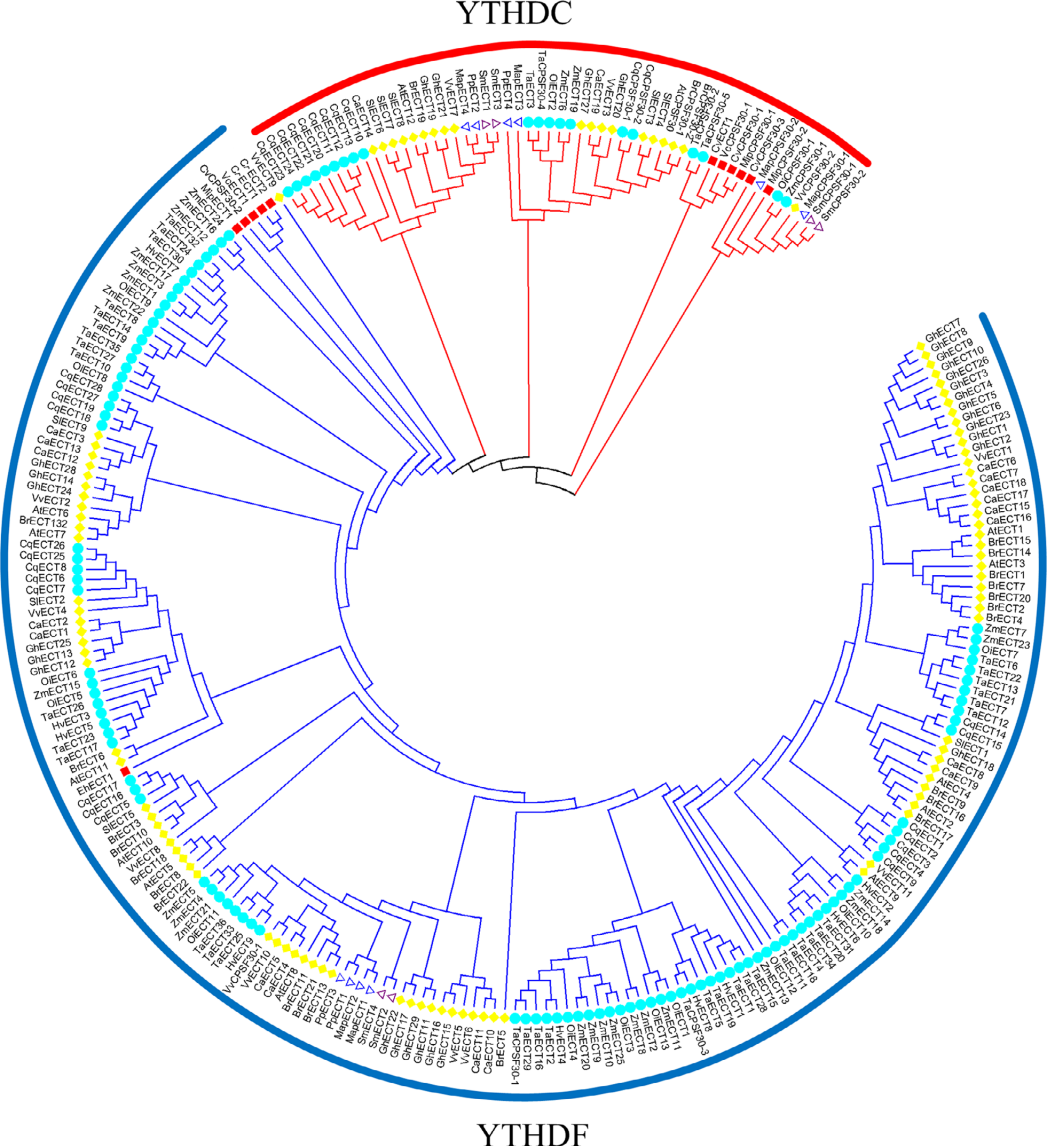
Phylogenetic analysis of 278 YTH domain‐containing proteins among 22 plant species (six dicotyledonous species, six monocotyledon species, one pteridophyte species, two moss species and seven algae species).

In this study, a total of 278 m6A readers in 22 representative plant species were identified by BLASTP and HMMER search (PF04146) (Table S3). On the basis of sequence similarity, YTH domain‐containing proteins can be classified into two distinct subfamilies: YTHDF and YTHDC (Patil *et al*., 2017; Scutenaire *et al*., 2018). YTHDF subfamily proteins primarily bind all m6A sites in mRNA, while YTHDC only binds certain nuclear‐enriched sites in mRNAs and non‐coding RNAs (Meyer and Jaffrey, 2017; Patil *et al*., 2017). In this study, the result of phylogenetic analysis showed that 55 YTH proteins could be classified into the YTHDC subfamily and 223 could be classified into the YTHDF subfamily (Figure 2). In conclusion, the identification, classification and characterization of these writers, erasers and readers will help to establish m6A regulatory pathways in plant biology.

## Function of m6A in plants

### The role of m6A in mRNA processing

*Arabidopsis* is an ideal model organism in which to study m6A RNA methylation due to the existence of a powerful gene knockout database. Only a little study has started on the exact mechanism and biological function of m6A modification or m6A‐related components in plants. Firstly, m6A has been revealed as one of the most important RNA modifications, playing a vital role in distinct steps of mRNA function, including mRNA degradation, stability, translation and miRNA processing in multiple species (Visvanathan and Somasundaram, 2018). For example, m6A modifications in 3′UTR and 5′UTR regions are positively correlated with gene expression, while m6A modifications in other regions result in lower gene expression in *Arabidopsis* (Luo *et al*., 2014). The ECT2 protein, as the YTHDF2 human homolog protein, not only regulates 3′UTR processing in the nucleus but also plays a critical role in promoting mRNA stability and controlling mRNA fate in the cytoplasm by binding m6A modifications (Lockhart, 2018b). Recent studies have found that 5′ UTR m6A modifications can affect protein translation. The first evidence for this came from studies of response to heat shock stress, which results in the redistribution of m6A, leading to increased m6A in the 5′ UTR and the promotion of protein translation under stress (Meyer *et al*., 2015). In *METTL3* mutants, there is a specific reduction in the translation of mRNAs containing m6A modifications in the 5′ UTR, but not in stop codons or 3′ UTR regions, further indicating that 5′ UTR m6A affects translation efficiency in cells (Meyer and Jaffrey, 2017). In addition, the differential localization of m6A modifications was found to result in distinct effects on translation mechanism. For example, cap‐binding factor eIF4E is necessary for translation initiation. 3′ UTR m6A of mRNAs can recruit ‘reader’ YTHDF1 and facilitate cap‐dependent translation, by recruiting the 43S pre‐initiation complex to the 5′ cap through an association between eIF4E, eIF4G and eIF3 (Wang *et al*., 2015b). Nevertheless, 5′‐UTR m6A in stress‐responsive genes encourages cap‐independent translation through the direct binding of eIF3 and later recruitment of the 43S ribosomal complex, which does not require eIF4E (Meyer *et al*., 2015). Moreover, the m6A eraser AtALKBH9B mediates mRNA silencing and decay processes (Chen *et al*., 2018).

### m6A function in plant development

The m6A modification is considered to play a critical role in plant embryonic development. The postembryonic expression level of m6A writer components, including MTA, MTB, FIP37, VIRILIZER and HAKAI, is reduced, resulting in the dramatic reduction of m6A. Reduced expression and knockout of these m6A writers produce distinct differentiation phenotypes, including an increase in the number of trichome branches, defective leaf initiation, the over‐proliferation of vegetative shoot apical meristem and finally embryonic lethality (Bodi *et al*., 2012; Růžička *et al*., 2017). Knockout of ECT2 protein increases trichome branching, suggesting that ECT2 is essential for regulating trichome branch development (Lockhart, 2018b; Scutenaire *et al*., 2018; Wei *et al*., 2018). Knockout of ALKBH10B caused a delay in flowering and also repressed vegetative growth, indicating that ALKBH10B‐mediated mRNA demethylation affected floral transition by influencing the stability of mRNA transcripts (Duan *et al*., 2017). Certain m6A‐modified genes play indispensable roles in regulating transcription factor activity in the callus, whereas other m6A‐modified genes are essential in plastid and thylakoid function in leaves (Li *et al*., 2014b). In addition, N6‐mAMP deaminase (MAPDA) catabolizes N6‐mAMP to IMP, which may be linked to root development, as the knockout of MAPDA leads to slightly reduced root growth (Chen *et al*., 2018).

### m6A role in stresses response

Growing evidence suggests that m6A is also involved in regulating response to various abiotic and biotic stresses. Diverse cellular stresses can result in a transcriptome‐wide redistribution of m6A, leading to an increase in the number of mRNAs with 5′ UTR m6A. 5′ UTR m6A directs the binding of eIF3 in a cap‐independent manner to promote translation initiation of mammalian mRNAs under heat stress, suggesting 5′ UTR m6A is vital in response to heat shock (Meyer *et al*., 2015). The m6A patterns are dynamic and 5‐30% of m6A peaks are altered under ultraviolet light, heat shock or interferon‐gamma, thereby influencing gene expression and splicing (Dominissini *et al*., 2012). In plants, research has uncovered the molecular mechanisms underlying m6A dynamics in response to stress. ECT1 and ECT2 are found to specifically interact with the stress response protein CIPK1 (Calcineurin B‐Like‐Interacting Protein Kinase1) and play an important role in the transmission of calcium signalling to the plant nucleus under various external stimuli (Ok *et al*., 2005). Although ECT2 lacks the YTH domain, it can strongly bind to cytosolic mRNA containing m6A modifications in 3′ untranslated regions. This ECT2‐mediated recognition of a plant‐specific m6A motif allows it to relocate mRNA to stress granules under heat stress (Scutenaire *et al*., 2018). It has also been confirmed that m6A levels are increased under biotic stresses. The demethylation activity of atALKBH9B decreased the level of m6A and may affect the infectivity of AMV (alfalfa mosaic virus) by interacting with the coat protein (CP). The m6A level in vRNAs of *Arabidopsis* was increased on alkbh9b mutation, which negatively regulated virus accumulation and systemic invasion (Martínez‐Pérez *et al*., 2017).

Transcriptome‐wide m6A mapping in differentiated callus and leaf from rice identified 8138 and 14 253 mRNAs with m6A modification, respectively (Li *et al*., 2014b). Such m6A sites are highly conserved between healthy unstressed cells and cells undergoing external stress, as well as between human and mouse cells (Dominissini *et al*., 2012; Fu *et al*., 2014; Jia *et al*., 2013). However, some m6A sites may also demonstrate species‐specific, cell‐specific or stress‐specific regulation (Meyer *et al*., 2012). For example, there is tissue specificity for the m6A sites in rice callus compared with leaves (Li *et al*., 2014b). Over 86% of transcripts in the *Arabidopsis* chloroplast and mitochondria are m6A methylated, with 64% and 79% of m6A methylated transcription showing differential tissue expression across leaves, flowers and roots (Wang *et al*., 2017). To understand the functional role of site‐specific m6A in mRNAs, it is necessary to determine m6A modifications in various species, cells and under external stresses. Furthermore, m6A modification appears a useful plant regulatory strategy to control gene expression, plant development and physiological processes (Lockhart, 2018b; Roignant and Soller, 2017).

## Evolution of m6A methylation components in the plant kingdom

**Figure 3:**
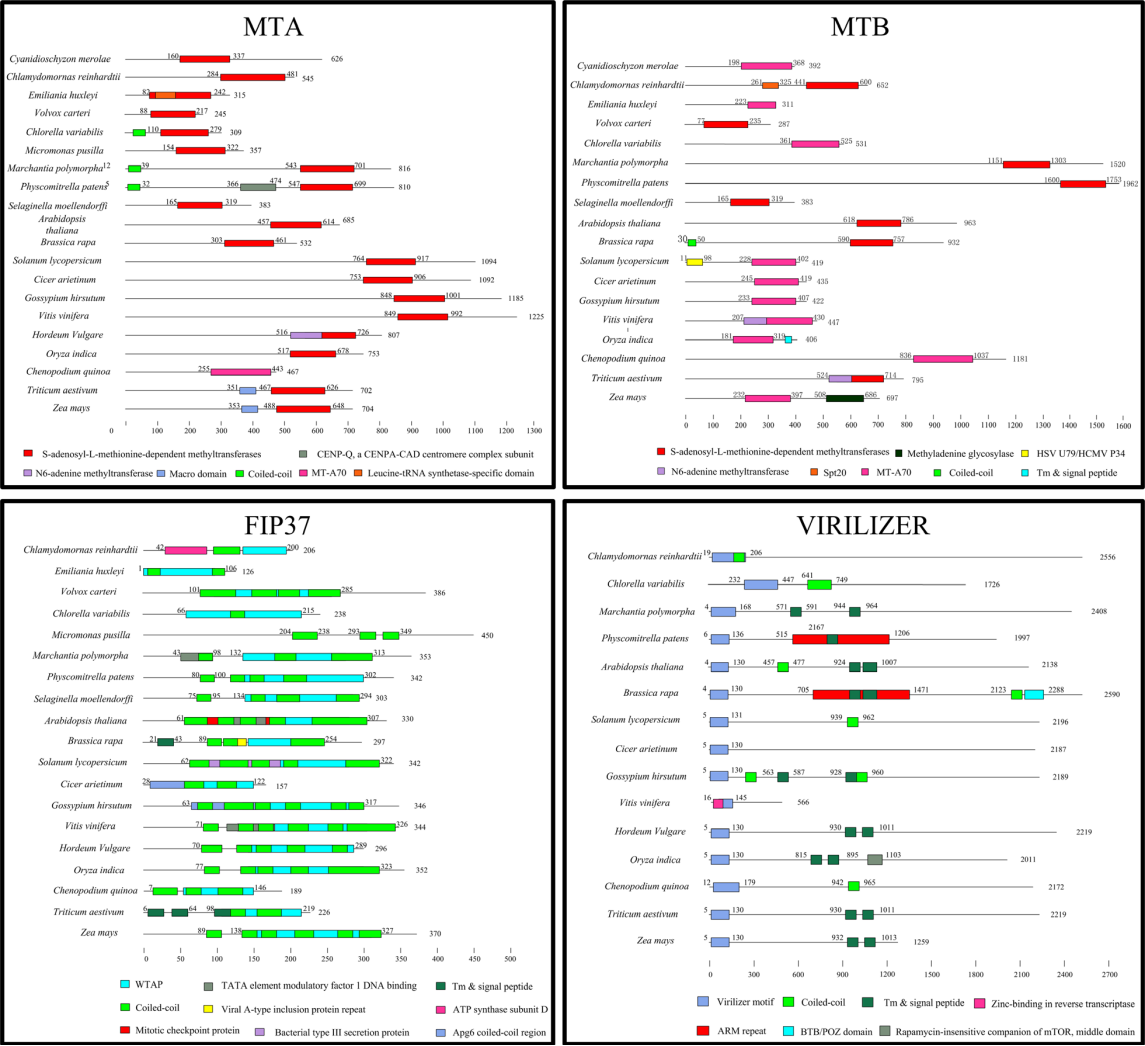
A comparison of functional domains in m6A writer proteins from various plant species. Schematic representation of the conserved domain structures of the four methyltransferases proteins, including MTA, MTB, FIP37 and VIRILIZER. Structural alignment of plant species showed that the SAM methyltransferase binding domain is at the C‐terminal region in MTA, and the SAM domain or MTA70 is also at the C‐terminal region in MTB. The WTAP domain is located internally in all FIP37 homologs, and the virilizer motif is at the N‐terminal region in all VIRILIZER homologs, respectively. Protein length is shown at the right of each protein schematic, and the location of each domain is indicated at the start and end of each motif box.

To understand the evolutionary history and relationship of m6A modification components, the abundance of m6A writer components in 22 representative plant species was analysed. The results showed that the number of m6A writer components was greater in higher plants than in lower plants, indicating that higher plants may require a more precise adjustment of m6A modifications to cope with complex and changing environments (Table S1). It was notable that m6A methyltransferases were not identified in *Ectocarpus siliculosus*. There was an absence of VIRILIZER and HAKAI‐related proteins from pteridophyte species and algae species, except for *Chlorella variabilis* and *Chlamydomona reinhardtii* (Table 1). *Chlamydomonas reinhardtii* is a unicellular green alga, and it evolved before the divergence of land plants. Consequently, these results might indicate that VIRILIZER and HAKAI underwent gene loss in these plant species. We speculate that pteridophyta and most algae species may have an alternative mechanism for m6A modification or another as of yet unknown protein instead of the two essential m6A writer components. However, there is little information available in current literature about this field, and we hope that m6A in lower plants will be extensively studied in the future. It is interesting that among the species studied, the evolutionarily complex allohexaploid wheat genome possessed the largest number of m6A writer components. At present, little research has been devoted to m6A writer components in plants. Identification of the m6A methyltransferase complex components in plants will contribute to the understanding of m6A function. To investigate the evolutionary relationships among the m6A writer domains, HMMER (https://www.ebi.ac.uk/Tools/hmmer/search/hmmscan) was used to predict the conserved protein structural domains in MTA, MTB, FIP37 and VIRILIZER. Details of the conserved domains identified are shown in Figure 3. The majority of MTA members possessed a SAM (S‐adenosyl methionine) methyltransferase binding domain, which was highly conserved in all species studied, except for *Chenopodium quinoa*. Furthermore, the MTB proteins in all species also contained a SAM methyltransferase binding domain or SAM binding site named MTA70, which is the domain responsible for methylation activity. All FIP37 proteins contained a WTAP (Wilms’ tumour 1‐associated protein) domain, and VIRILIZER proteins contained a virilizer motif in all species. The results showed that the protein sequences and domain structure of m6A writer components were highly conserved in all plants studied (Figure 3).

**Figure 4:**
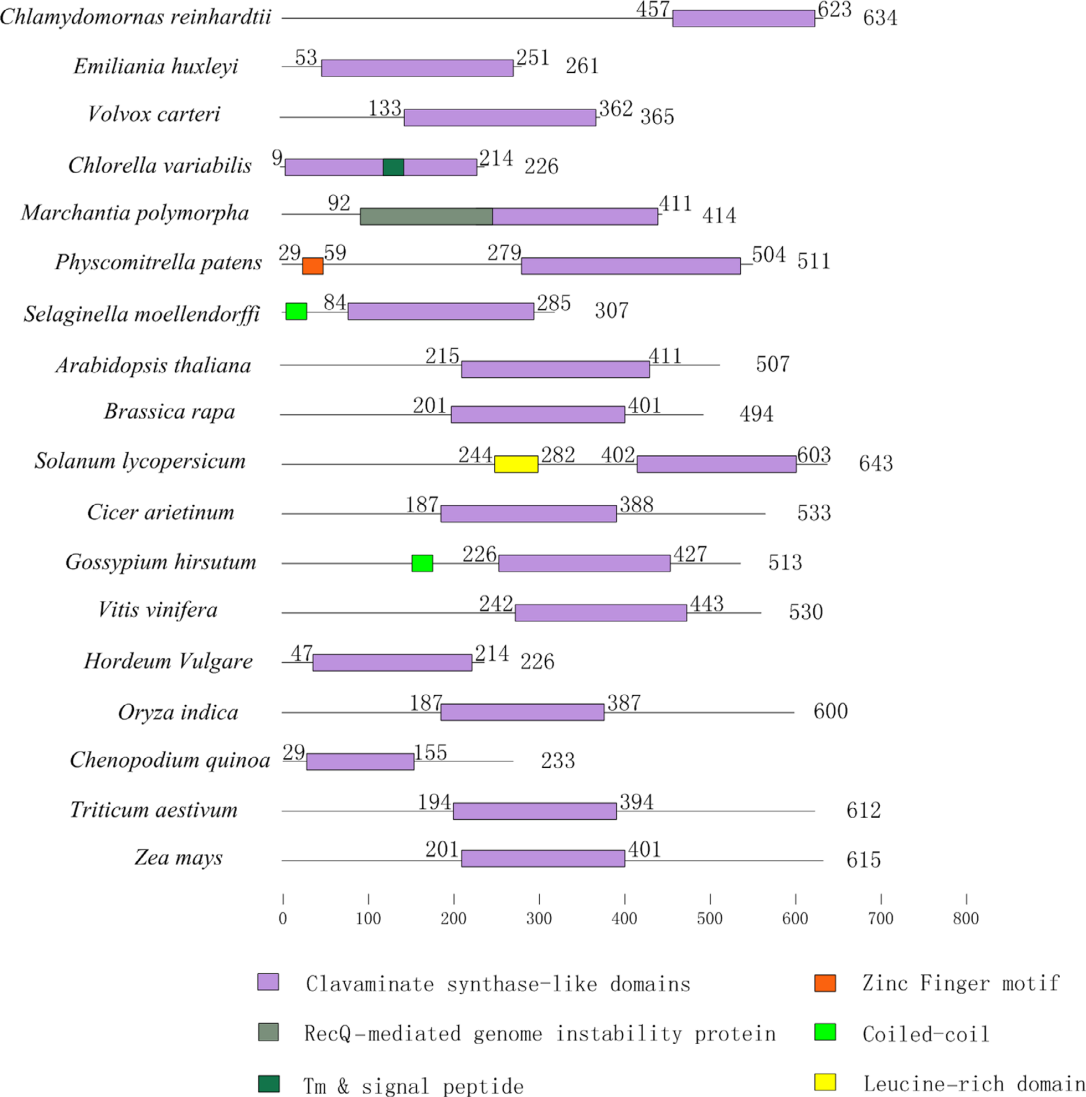
Schematic of conserved functional domains of m6A erasers, termed as AlkBH proteins, in 18 plant species. The purple box represents the clavaminate synthase‐like domain. All plant species show a highly conserved domain structure. Protein length is shown at the right of each protein schematic, and the location of the clavaminate synthase‐like domain is indicated at the start and end of each motif box.

Angiosperm plants appear to have the most abundant numbers of AlkBH proteins, followed by pteridophyte species, and lastly bryophyte species. The number of AlkBH proteins in algae was particularly low when compared to that of other plant species, except for *Emiliania huxleyi*. In particular, a total of 29 and 27 AlkBH proteins were identified in *Triticum aestivum* and *Chenopodium quinoa*, respectively (Table S2). This main difference between the species studied was that genomes of land plants have numerous AlkBH proteins, while algae may have only a single copy. It would be important to further explore how the AlkBH protein class expanded during the process of plant evolution from lower unicellular organisms to higher flowering plants. All plant AlkBH family members are highly similar to each other and have the clavaminate synthase‐like domain. It is worth noting that *Physcomitrella patens* has one zinc finger motif and *Solanum lycopersicum* has one leucine‐rich domain, respectively, suggesting that they may also act as transcription factors to regulate gene expression (Figure 4).

m6A may retain certain conserved functions during evolution. Such orthologous proteins are generally known to perform analogous biological functions and are widely distributed in diverse species (Schlicker *et al*., 2006). Phylogenetic analysis is a rapid and relatively accurate method to identify orthologous proteins (Bauwens *et al*., 2018; Jensen *et al*., 2018). Consequently, we constructed a phylogenetic tree for AlkBH proteins based on sequence similarity using MEGA6.0 and the neighbour‐joining (NJ) method was adopted with 1000 bootstrap replications (Kumar *et al*., 2008). Proteins were considered orthologous to *Arabidopsis* atALKBH9 and atALKBH10 if they possessed a function similar to m6A demethylation (Figure [Link], [Link], [Link]). For example, 12 ALKBH proteins were identified in rice, with OiALKBH5B, OiALKBH6B and OiALKBH12B classed as potential m6A erasers. In addition, 29 ALKBH proteins were identified in wheat, with TaALKBH4B, TaALKBH6B and TaALKBH29B classed as m6A erasers. All postulated m6A demethylases in 21 species are listed in Table 1. Currently, there are no m6A erasers identified in rice, although we found OsALKBH1 as a potential member of this class (Liang *et al*., 2018). Aravind and colleagues hypothesize that EGL‐9, as a homolog of AlkB, might be involved in RNA demethylation in plant RNA viruses (Aravind and Koonin, 2001). The findings in this study may be useful to researchers attempting to understand m6A mechanisms.

Plants have more YTH domain proteins than other eukaryotes, including yeast, human and other mammals (Meyer and Jaffrey, 2017). For instance, 13 YTH domain proteins named ECT1‐12 and CPSF30 were found in *Arabidopsis*, which is significantly higher than that of the 5 YTH proteins identified in mammals (Meyer and Jaffrey, 2017; Wei *et al*., 2018). The results showed that YTH proteins were widespread in land plants. The number of YTH proteins in angiosperm plants was particularly high, with 41, 30 and 29 YTH proteins found in *Triticum aestivum*,* Chenopodium quinoa* and *Gossypium hirsutum*, respectively. Fewer YTH proteins were identified in pteridophyta, bryophytes and algae (Table S3), and their biological importance remains unknown. It is worth noting that the YTH domain protein Mmi1 in fission yeast (*Schizosaccharomyces pombe*) does not bind to m6A but can recognize a specific nucleotide sequence, indicating that not all YTH domain related to bind m6A (Wang *et al*., 2015a).

**Figure 5:**
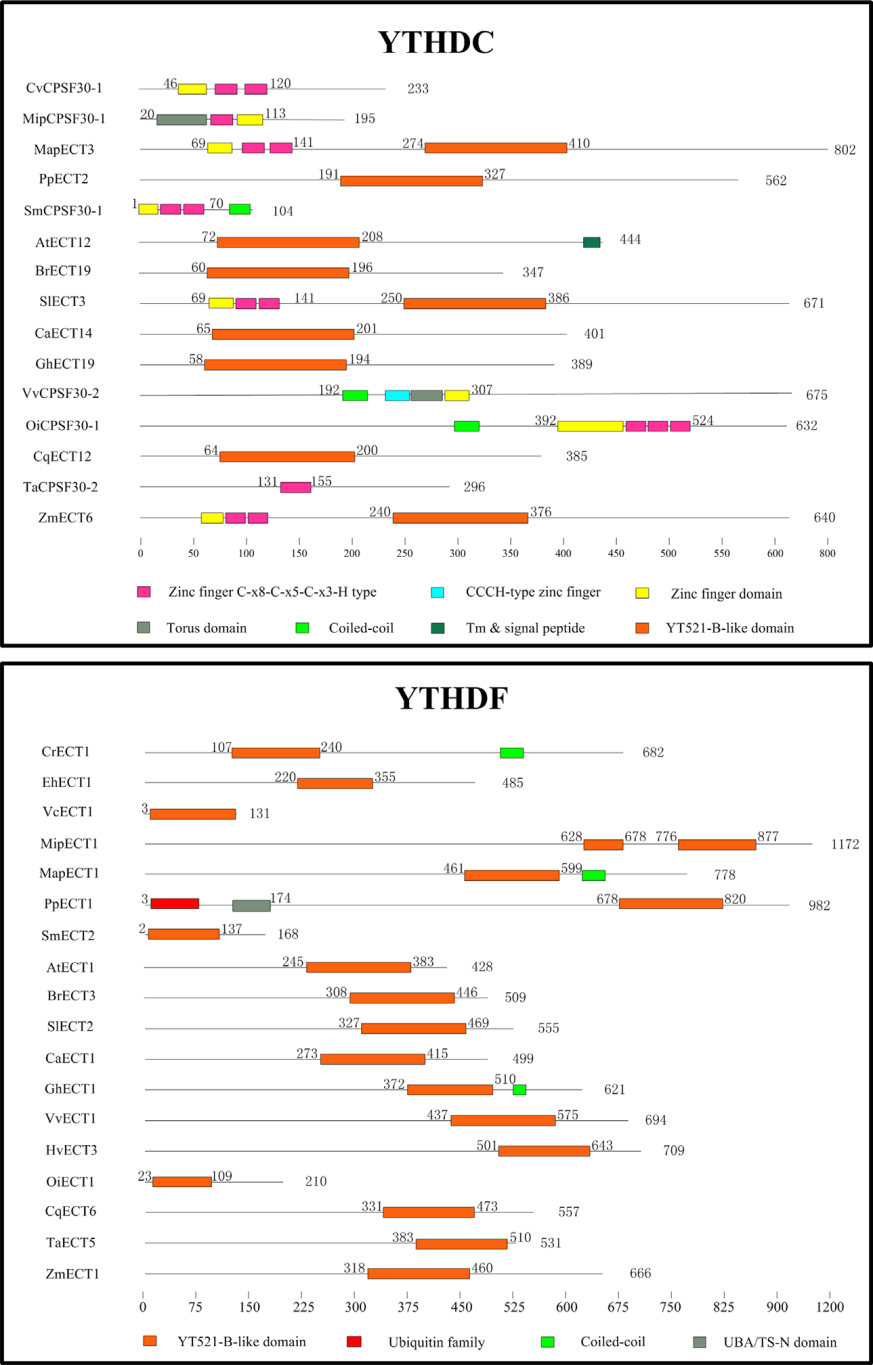
The comparison of functional domains in m6A readers from various plant species. YTH proteins can be classified into two distinct subfamilies: YTHDF and YTHDC. The orange box represents the highly conserved YTH domain. Protein length is shown at the right of each protein schematic, and the location of each domain is indicated at the start and end of each motif box.

AtCPSF30 is a member of the plant polyadenylation complex, which belongs to the YTHDC subfamily in *Arabidopsis* (Addepalli and Hunt, 2007). All plant YTHDC subfamily proteins contain the YTH domain or highly conserved zinc fingers (Figure 5). No YTHDC protein was found in five algae studied (*Cyanidioschyzon merolae, Emiliania huxleyi, Volvox carteri, Ectocarpus siliculosus and Chlamydomonas reinhardtii*), suggesting no gene duplication event of YTHDC occurred during the evolution process of most algae. It is worth noting that two monocotyledons (*Sorghum bicolor* and *Hordeum vulgare*) lack YTHDC proteins, while all dicotyledon, pteridophyta and bryophytes species have one or more members. We presume that the YTHDC subfamily may have been lost in the evolution of different monocotyledons species. Some studies have reported that YTHDC1 is the dominant protein to regulate alternative splicing in endogenous transcripts (Zhang *et al*., 2010). Importantly, only YTHDC1 could bind to 76 m6A sites on XIST mRNA, which plays the core role in silencing genes in female cells. YTHDC1 would promote XIST function to induce gene repression on the X chromosome (Patil *et al*., 2016). The other reader protein, YTHDC2, increases the translation efficiency of HIF1α mRNA through its helicase function (Tanabe *et al*., 2016).

One main functional YTH domain was identified in the YTHDF subfamily members of all plant species. It is highly conserved and predominantly cytoplasmic (Figure 5). The YTH domain in wheat is located at the N‐terminal, suggesting that TaECT5 may bind m6A in mRNA from the translatable pool and move to processing bodies. In contrast, The YTH domain in *Selaginella moellendorffi* and *Oryza indica* is located at the C‐terminal, suggesting that SmECT2 and OiECT1 selectively bind m6A (Wang *et al*., 2014). All species contain at least one YTHDF subfamily member, indicating that the common ancestor of plants may have undergone gene duplication, with these duplicated copies further evolving new features.

ECT2, ECT3 and ECT4 were confirmed as m6A readers in *Arabidopsis* and can recognize m6A sites. Another protein associated with the YTH domain is CPSF30 (30 kDa cleavage and polyadenylation specificity factor 30) in *Arabidopsis* (Arribas‐Hernández *et al*., 2018). CPSF30 localizes to the nucleus and plays an important role in response to external stimuli by regulating the splicing of the 3′ end of mRNA involved in the salicylic acid pathway of *Arabidopsis* (Bruggeman *et al*., 2014). Proteins orthologous to ECT2/3/4 and CPSF30 of *Arabidopsis* have been identified in 21 other species through the construction of a phylogenetic tree (Figure [Link], [Link], [Link], [Link]). 41 YTH proteins were identified in wheat, with only eight proteins found as potential m6A readers. 14 YTHs were identified in rice, with three proteins found as potential m6A readers (Table 1). These orthologous proteins are considered to have a function similar to their orthologues in *Arabidopsis*, but it is still not clear whether these proteins actually act as m6A readers in plants. In conclusion, m6A modification components have been present within the plant common ancestor since early plant evolution, and the protein domains associated with each of these components were highly conserved across algae, pteridophyta and angiosperm. Certain protein components appear to have been lost during the evolutionary process. Further insight into the evolutionary relationship and duplicative expansion of m6A components between lower and higher plants should provide a greater understanding of their function.

## Conclusion and future perspectives

In this study, we systematically reviewed the structure, composition, function and evolution of m6A regulatory machinery in plants. Such sequence comparative investigations on these m6A related components will aid in understanding the dynamic processes of m6A modification, as well as its functional roles. This research will further deepen our understanding of how m6A exerts RNA epigenetic regulation in plants. Recently, more efficient technology has been developed to detect m6A modifications and characterize m6A function. Two methods, MeRIP‐Seq and m6A‐seq, combine highly specific m6A antibody immunoblotting and high‐throughput deep sequencing to efficiently and accurately determine the methylated transcripts (Dominissini *et al*., 2012; Meyer *et al*., 2012). In addition, the newer m6A individual‐nucleotide‐resolution cross‐linking and immunoprecipitation (miCLIP) technique can easily and sensitively detect m6A at single‐nucleotide resolution (Linder *et al*., 2015). In 2017, a chemical proteomics approach was developed, which relies upon photo‐cross‐linking with RNA probes containing synthetic diazirine, to explore RNA–protein interactions controlled by m6A (Arguello *et al*., 2017). The replacement of oxygen at the 4‐position of deoxythymidine triphosphate with larger atoms (sulphur and selenium) weakened the ability of m6A to base pair. This silent modification could be detected in FTO through next‐generation sequencing. Using this method, two closely situated m6A sites could be detected at single‐nucleotide resolution (Hong *et al*., 2018). Furthermore, the AthMethPre web server, an integrated R application PEA, m6ASNP web server and m6AVar database were developed to predict the target m6A modification sites (Jiang *et al*., 2018; Xiang *et al*., 2016; Zhai *et al*., 2018). Zhou developed a computational pipeline termed AutoCirc, which identified thousands of cell‐specific m6A modifications in circRNAs (Zhou *et al*., 2017). These methods have enabled in‐depth studies of m6A methylation events and will be useful to analyse the role of m6A in the binding of specific transcripts in plants.

Despite such progress, further work is required to fully understand m6A modification processes and function. Firstly, the identity of m6A writers, erasers and readers should be verified, which will aid in understanding how the methylation level on numerous types of RNA molecules is regulated in plant species. Secondly, the expression pattern of m6A should be carefully validated, as various m6A patterns were obtained from different members of the same gene family, resulting in the differential transport or translation of mRNA. Thirdly, although knowledge on the function and molecular mechanism of m6A modification in plants is gradually increasing, the majority of studies are conducted in the model system *Arabidopsis*, while little research effort is directed towards crops. It is currently unknown how m6A modification regulates organ formation, cell division, growth and development, as well as stress response in crops, especially in the staple crops worldwide, including wheat, barley and rice. Future studies on m6A modifications in crops could provide valuable information for the further improvement of seed yield and stress tolerance in crops.

## Competing interests

The authors declare that they have no competing interests.

## Author contributions

X.J.N. involved in conceptualization; H.Y. acquired, analysed and interpreted the data; H.Y. and Z.J.Y involved in writing the original article and draft preparation; W.N.S. wrote, reviewed and edited the manuscript; X.J.N. and H.Y. involved in funding acquisition.

## Supporting information


**Figure S1** Orthologous protein of *Arabidopsis* atALKBH9 and atALKBH10 were identified by phylogenetic analysis among *Micromonas pusilla*,* Emiliania huxleyi*,* Volvox carteri*,* Ectocarpus siliculosus*,* Chlorella variabilis*,* Selaginella moellendorffi*, and *Chlamydomonas reinhardtii*.

**Figure S2** Orthologous protein of *Arabidopsis* atALKBH9 and atALKBH10 were identified by phylogenetic analysis among *Physcomitrella patens, Cicer arietinum, Solanum lycopersicum, Vitis vinifera, Marchantia polymorpha, Brassica rapa* and *Chenopodium quinoa*.

**Figure S3** Orthologous protein of *Arabidopsis* atALKBH9 and atALKBH10 were identified by phylogenetic analysis among *Zea mays, Triticum aestivum, Sorghum bicolor, Oryza indica* and *Hordeum vulgare*.

**Figure S4** Orthologous protein of *Arabidopsis* ECT2, ECT3 and ECT4 were identified by phylogenetic analysis among *Zea mays, Micromonas pusilla*,* Emiliania huxleyi*,* Volvox carteri*,* Gossypium hirsutum, Ectocarpus siliculosus*,* Chlorella variabilis* and *Chlamydomonas reinhardtii*.

**Figure S5** Orthologous protein of *Arabidopsis* ECT2, ECT3 and ECT4 were identified by phylogenetic analysis among *Selaginella moellendorffi*,* Physcomitrella patens, Cicer arietinum, Solanum lycopersicum, Marchantia polymorpha* and *Brassica rapa*.

**Figure S6** Orthologous protein of *Arabidopsis* ECT2, ECT3 and ECT4 were identified by phylogenetic analysis among *Vitis vinifera, Chenopodium quinoa, Triticum aestivum* and *Sorghum bicolor*.

**Figure S7** Orthologous protein of *Arabidopsis* ECT2, ECT3 and ECT4 were identified by phylogenetic analysis among *Oryza indica* and *Hordeum vulgare*.

**Table S1** Accession numbers, name codes and amino acid sequences of m6A writers from plant species.

**Table S2** Accession numbers, name codes and amino acid sequences of m6A erasers from plant species.

**Table S3** Accession numbers, name codes and amino acid sequences of m6A readers from plant species.
