# Supplementary figures and images for: N6‐methyladenosine regulatory machinery in plants: composition, function and evolution
Source: Plant Biotechnol J. 2019 May 21;17(7):1194–208. doi: 10.1111/pbi.13149 (PMC6576107; doi:10.1111/pbi.13149)

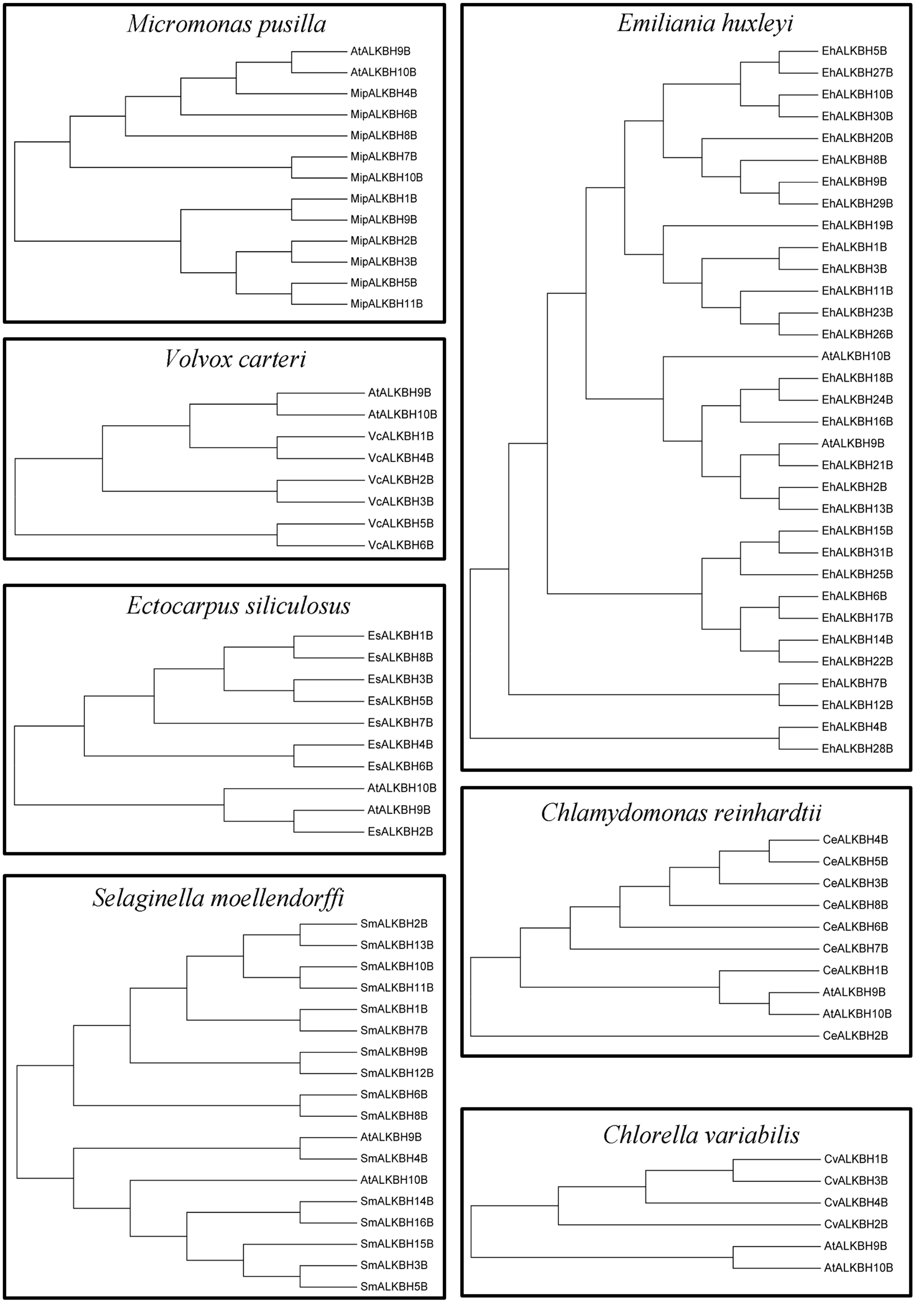

Supplement: Supplementary file 1 — Figure S1 Orthologous protein of Arabidopsis atALKBH9 and atALKBH10 were identified by phylogenetic analysis among Micromonas pusilla, Emiliania huxleyi, Volvox carteri, Ectocarpus siliculosus, Chlorella variabilis, Selaginella moellendorffi, and Chlamydomonas reinhardtii. [file PBI-17-1194-s008.tif]

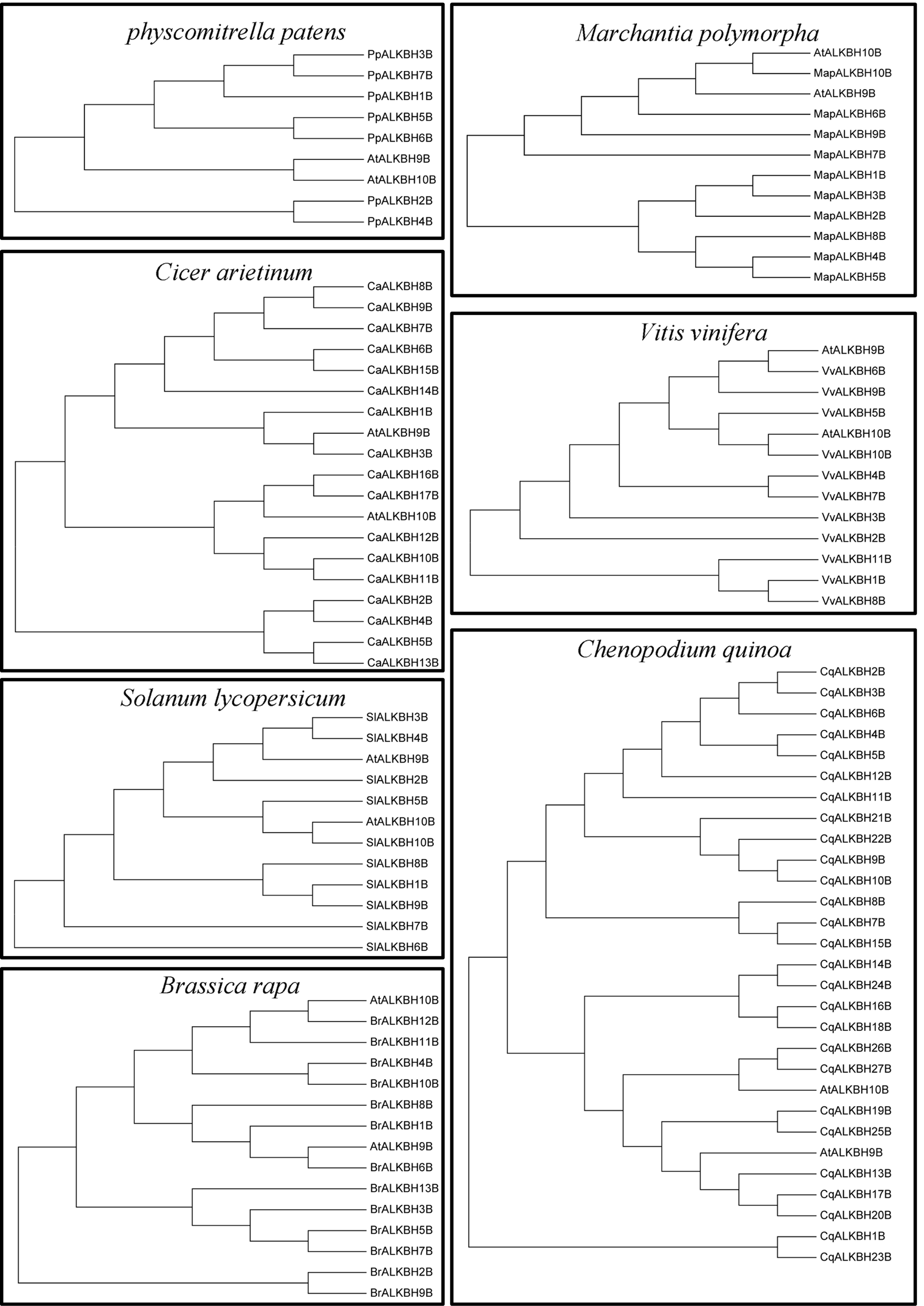

Supplement: Supplementary file 2 — Figure S2 Orthologous protein of Arabidopsis atALKBH9 and atALKBH10 were identified by phylogenetic analysis among Physcomitrella patens, Cicer arietinum, Solanum lycopersicum, Vitis vinifera, Marchantia polymorpha, Brassica rapa and Chenopodium quinoa. [file PBI-17-1194-s007.tif]

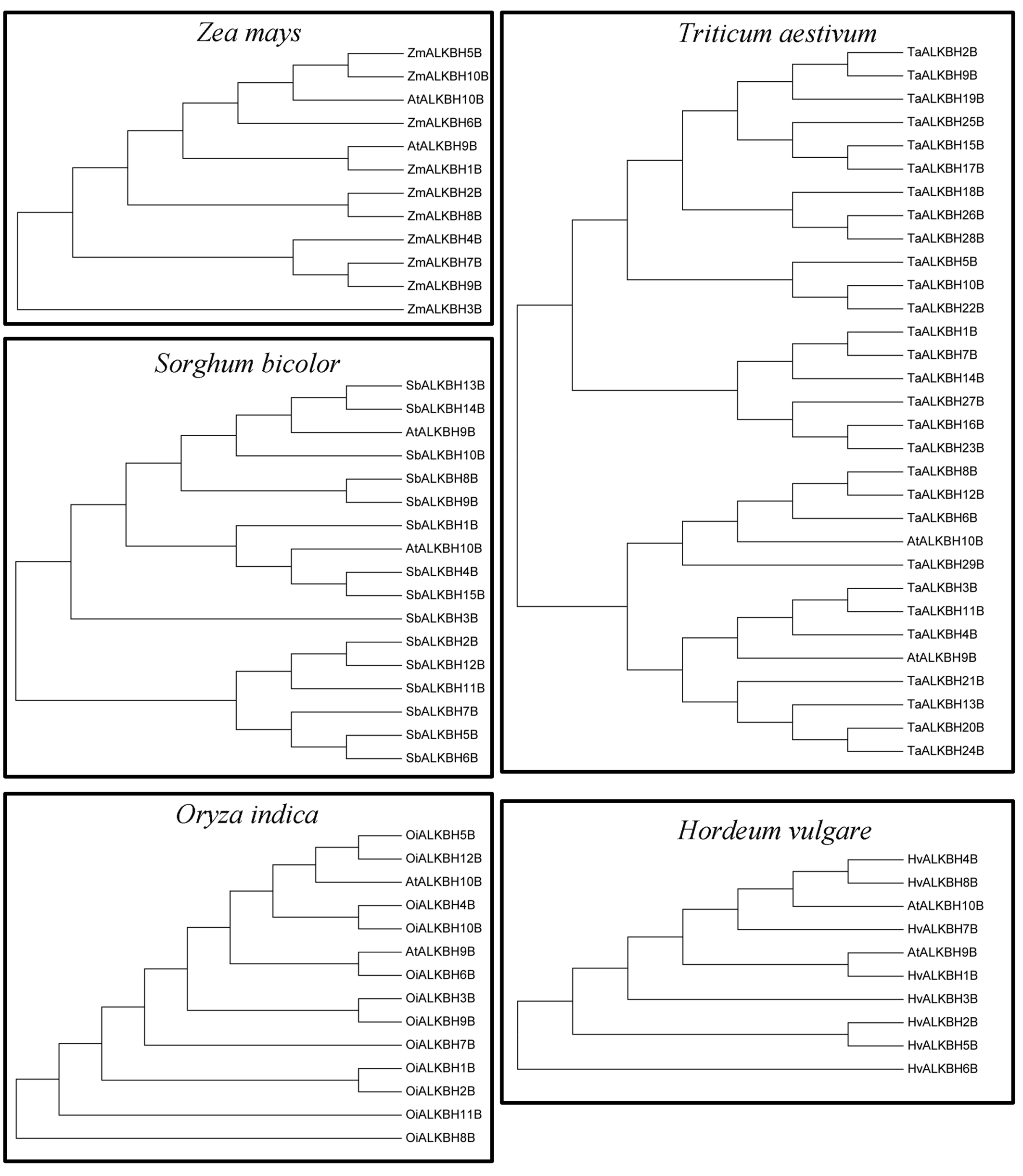

Supplement: Supplementary file 3 — Figure S3 Orthologous protein of Arabidopsis atALKBH9 and atALKBH10 were identified by phylogenetic analysis among Zea mays, Triticum aestivum, Sorghum bicolor, Oryza indica and Hordeum vulgare. [file PBI-17-1194-s006.tif]

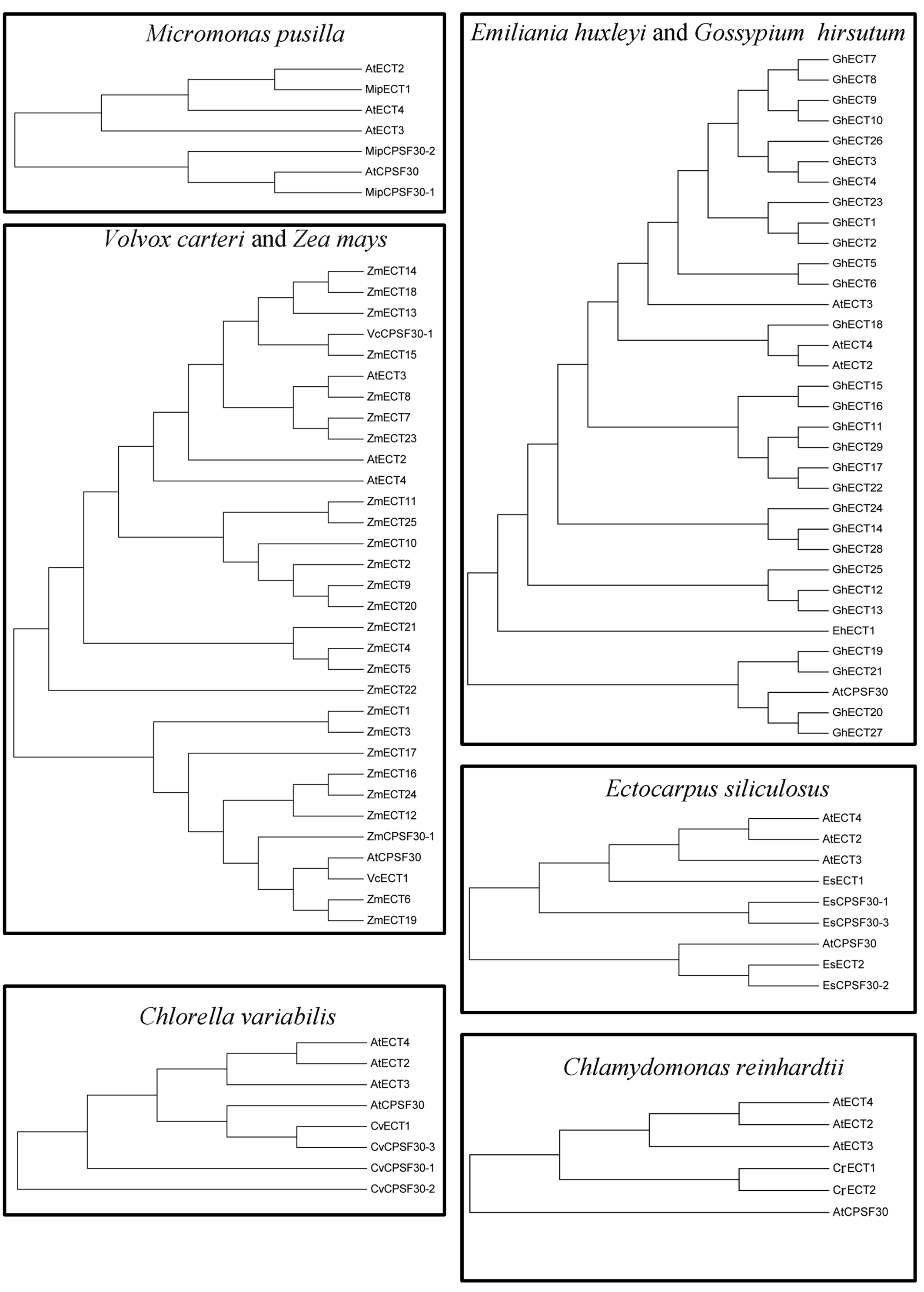

Supplement: Supplementary file 4 — Figure S4 Orthologous protein of Arabidopsis ECT2, ECT3 and ECT4 were identified by phylogenetic analysis among Zea mays, Micromonas pusilla, Emiliania huxleyi, Volvox carteri, Gossypium hirsutum, Ectocarpus siliculosus, Chlorella variabilis and Chlamydomonas reinhardtii. [file PBI-17-1194-s010.tif]

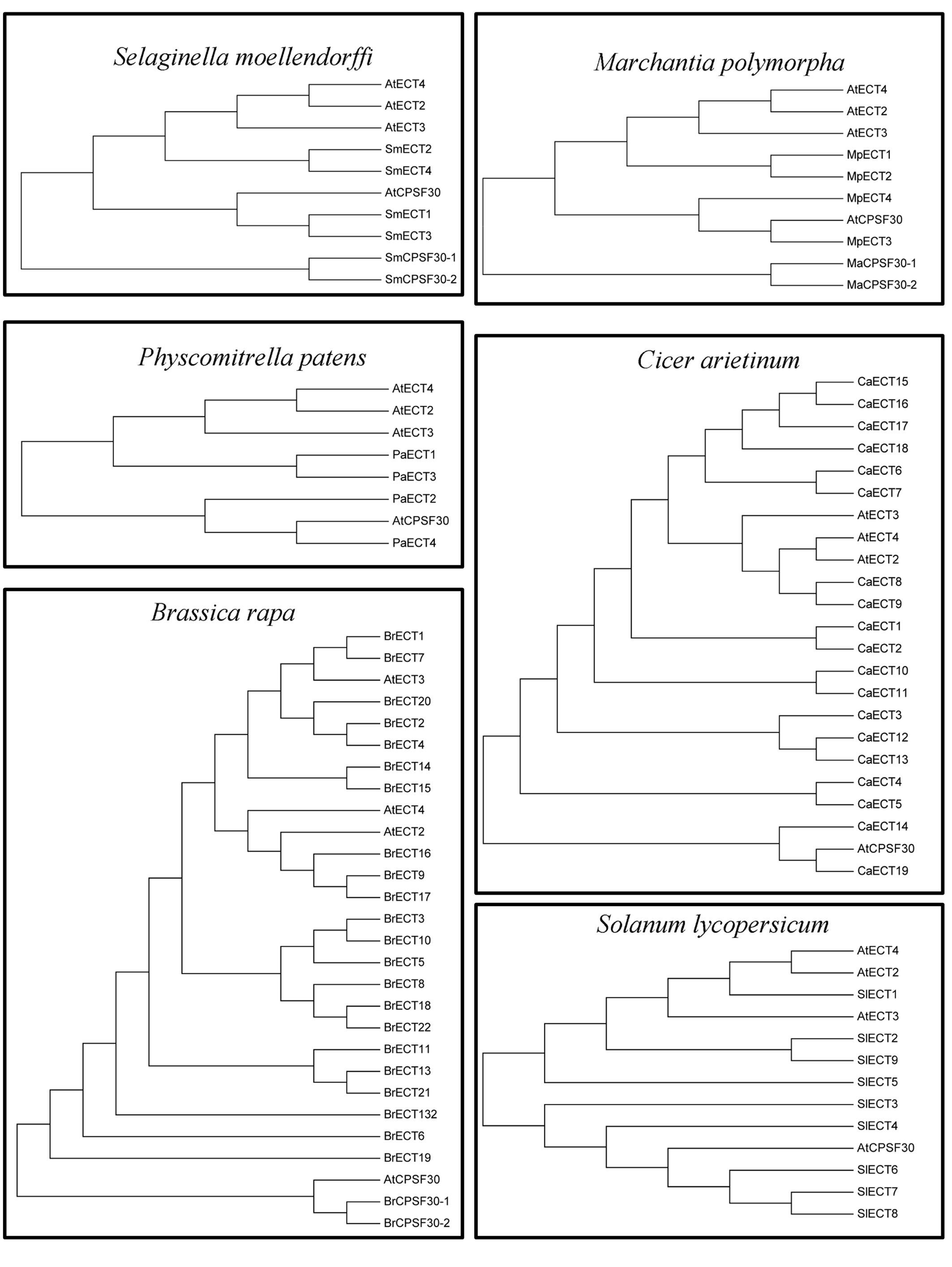

Supplement: Supplementary file 5 — Figure S5 Orthologous protein of Arabidopsis ECT2, ECT3 and ECT4 were identified by phylogenetic analysis among Selaginella moellendorffi, Physcomitrella patens, Cicer arietinum, Solanum lycopersicum, Marchantia polymorpha and Brassica rapa. [file PBI-17-1194-s001.tif]

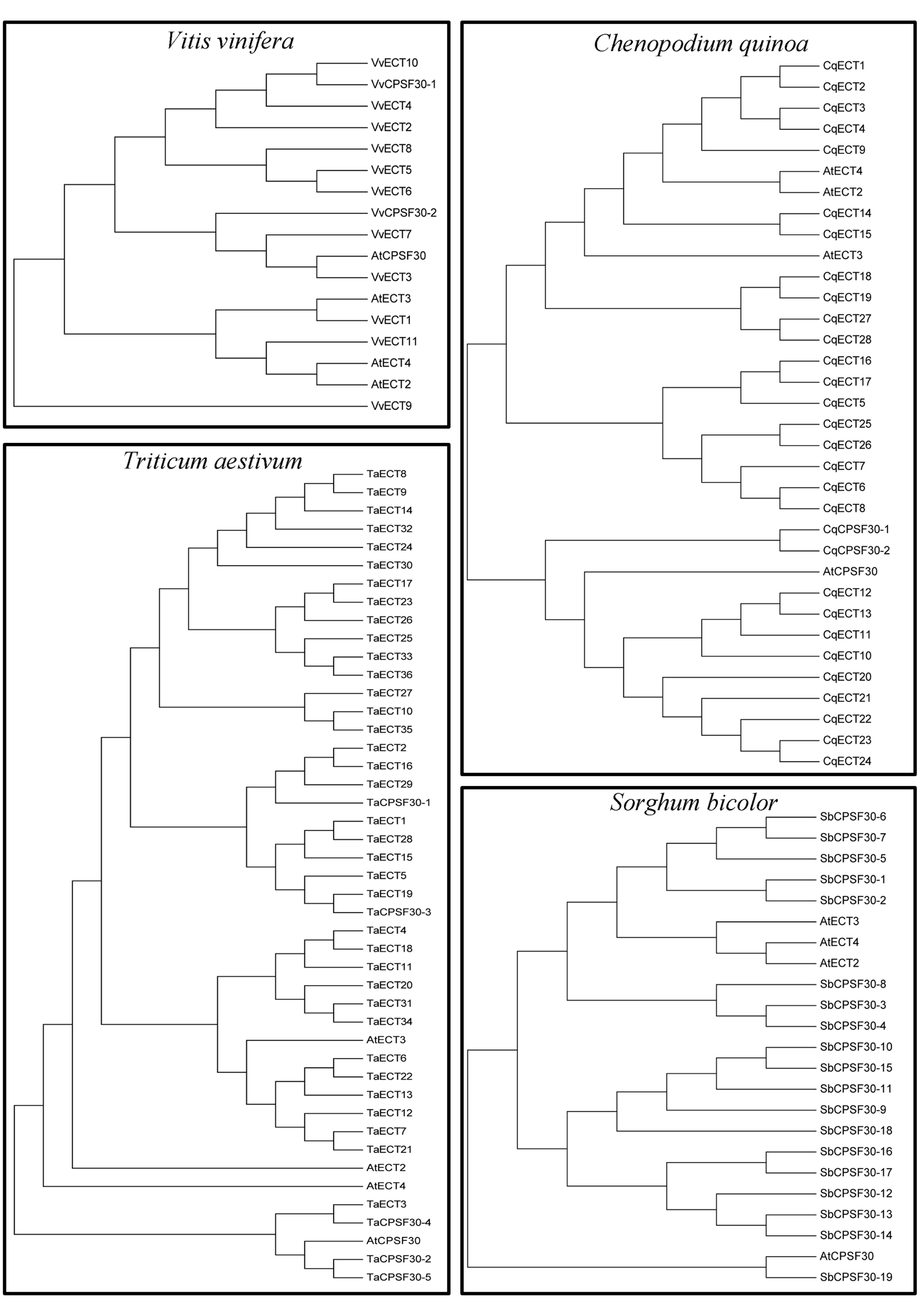

Supplement: Supplementary file 6 — Figure S6 Orthologous protein of Arabidopsis ECT2, ECT3 and ECT4 were identified by phylogenetic analysis among Vitis vinifera, Chenopodium quinoa, Triticum aestivum and Sorghum bicolor. [file PBI-17-1194-s002.tif]

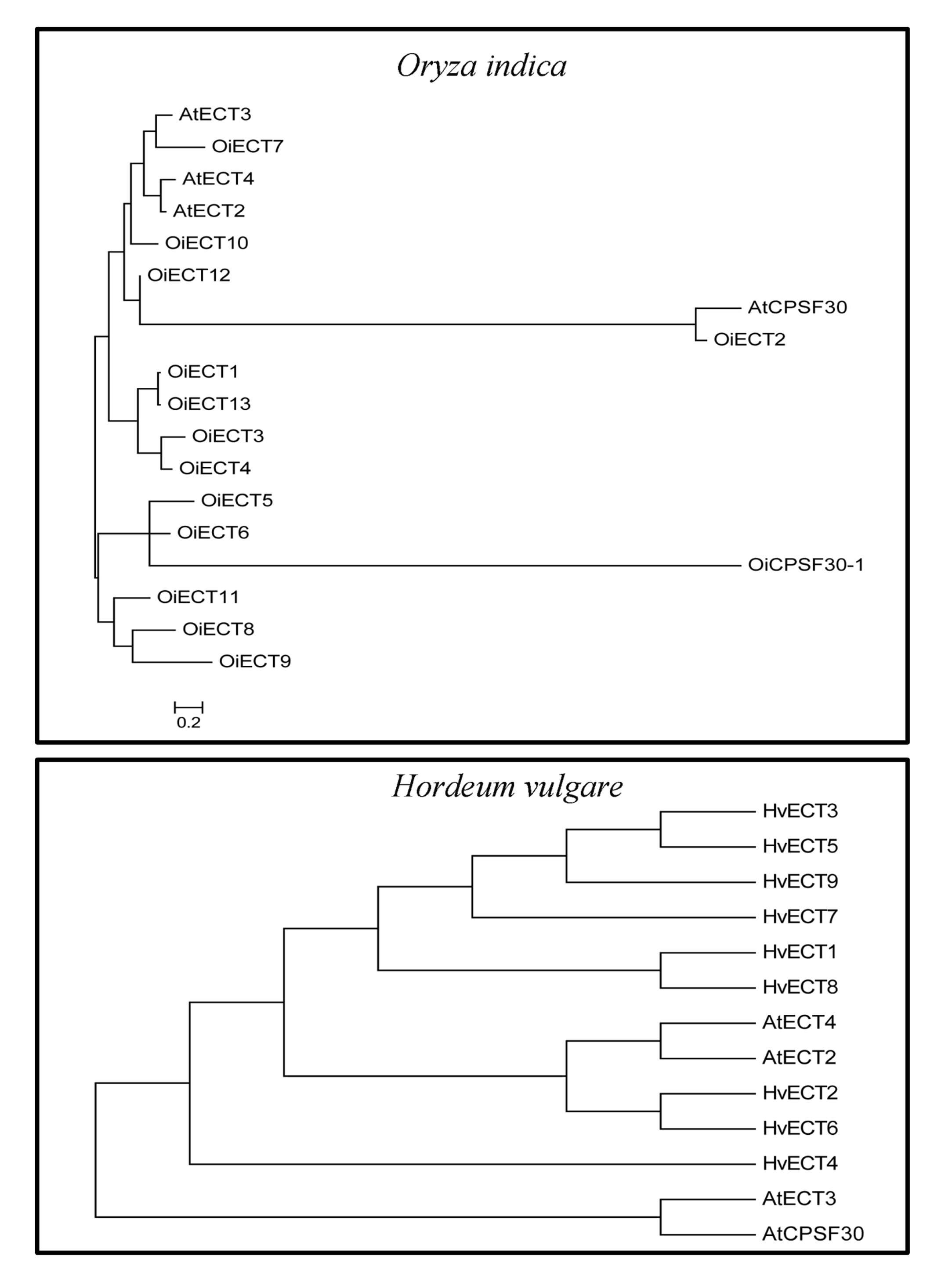

Supplement: Supplementary file 7 — Figure S7 Orthologous protein of Arabidopsis ECT2, ECT3 and ECT4 were identified by phylogenetic analysis among Oryza indica and Hordeum vulgare. [file PBI-17-1194-s003.tif]
