# Supplementary material for: N6‐methyladenosine regulatory machinery in plants: composition, function and evolution
Source: Plant Biotechnol J. 2019 May 21;17(7):1194–208. doi: 10.1111/pbi.13149 (PMC6576107; doi:10.1111/pbi.13149)
Supplement: Supplementary file 11 — Supplementary Legends [file PBI-17-1194-s009.docx]

**Supplementary materials**

**Figure S1** Orthologous protein of *Arabidopsis* atALKBH9 and atALKBH10 were identified by phylogenetic analysis among *Micromonas pusilla*, *Emiliania huxleyi*, *Volvox carteri*, *Ectocarpus siliculosus*, *Chlorella variabilis*, *Selaginella moellendorffi*, and *Chlamydomonas reinhardtii*.

**Figure S2** Orthologous protein of *Arabidopsis* atALKBH9 and atALKBH10 were identified by phylogenetic analysis among *Physcomitrella patens, Cicer arietinum, Solanum lycopersicum, Vitis vinifera, Marchantia polymorpha, Brassica rapa* and *Chenopodium quinoa*.

**Figure S3** Orthologous protein of *Arabidopsis* atALKBH9 and atALKBH10 were identified by phylogenetic analysis among *Zea mays, Triticum aestivum, Sorghum bicolor, Oryza indica* and *Hordeum vulgare*.

**Figure S4** Orthologous protein of *Arabidopsis* ECT2, ECT3 and ECT4 were identified by phylogenetic analysis among *Zea mays, Micromonas pusilla*, *Emiliania huxleyi*, *Volvox carteri*, *Gossypium hirsutum, Ectocarpus siliculosus*, *Chlorella variabilis* and *Chlamydomonas reinhardtii*.

**Figure S5** Orthologous protein of *Arabidopsis* ECT2, ECT3 and ECT4 were identified by phylogenetic analysis among *Selaginella moellendorffi*, *Physcomitrella patens, Cicer arietinum, Solanum lycopersicum, Marchantia polymorpha* and *Brassica rapa*.

**Figure S6** Orthologous protein of *Arabidopsis* ECT2, ECT3 and ECT4 were identified by phylogenetic analysis among *Vitis vinifera, Chenopodium quinoa, Triticum aestivum* and *Sorghum bicolor.*

**Figure S7** Orthologous protein of *Arabidopsis* ECT2, ECT3 and ECT4 were identified by phylogenetic analysis among *Oryza indica* and *Hordeum vulgare*.

**Table S1** Accession numbers, name codes and amino acid sequences of m6A writers from plant species.

**Table S2** Accession numbers, name codes and amino acid sequences of m6A erasers from plant species.

**Table S3** Accession numbers, name codes and amino acid sequences of m6A readers from plant species.


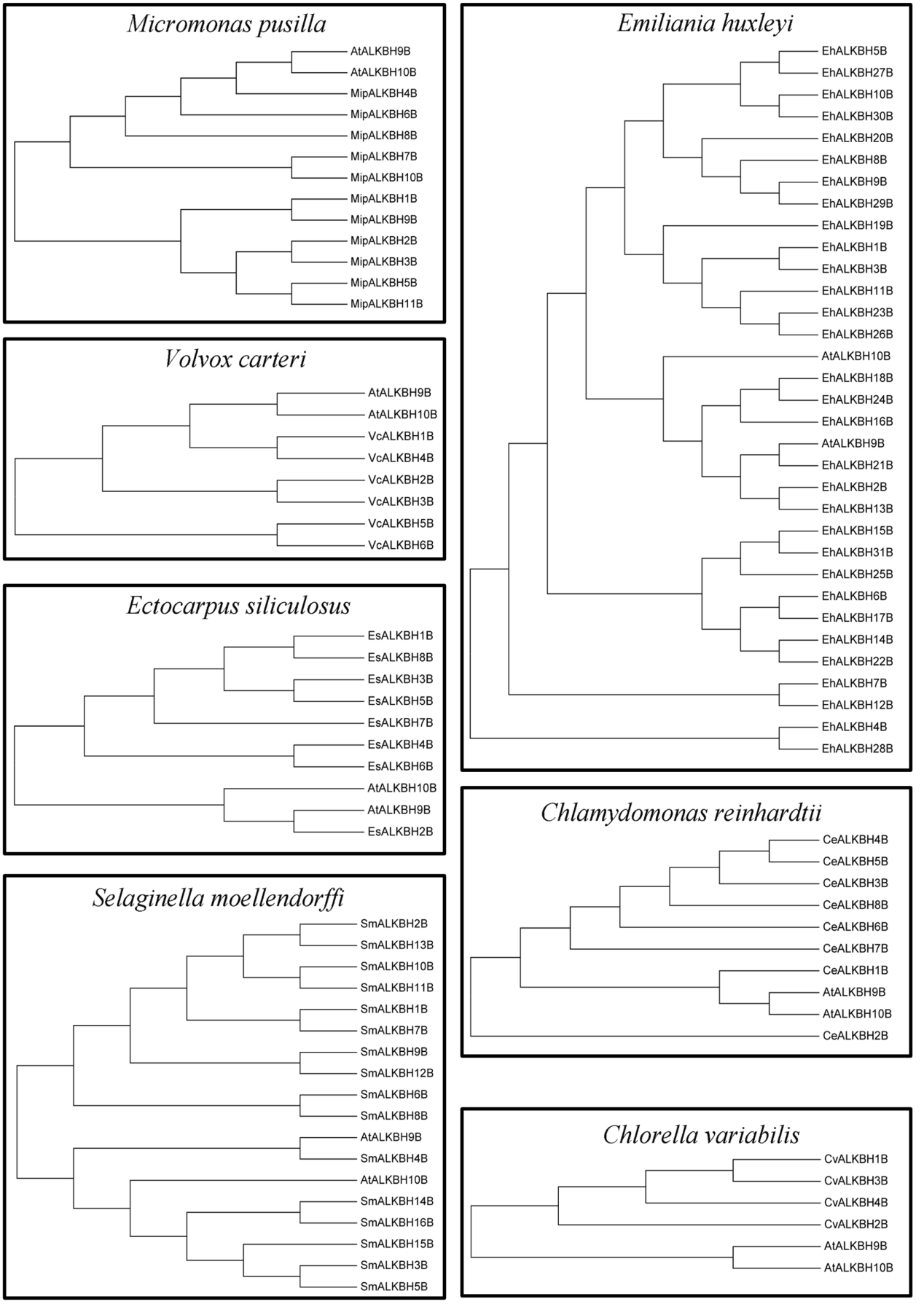


Figure S1. Orthologous protein of *Arabidopsis* atALKBH9 and atALKBH10 were identified by phylogenetic analysis among *Micromonas pusilla*, *Emiliania huxleyi*, *Volvox carteri*, *Ectocarpus siliculosus*, *Chlorella variabilis*, *Selaginella moellendorffi*, and *Chlamydomonas reinhardtii*.

**
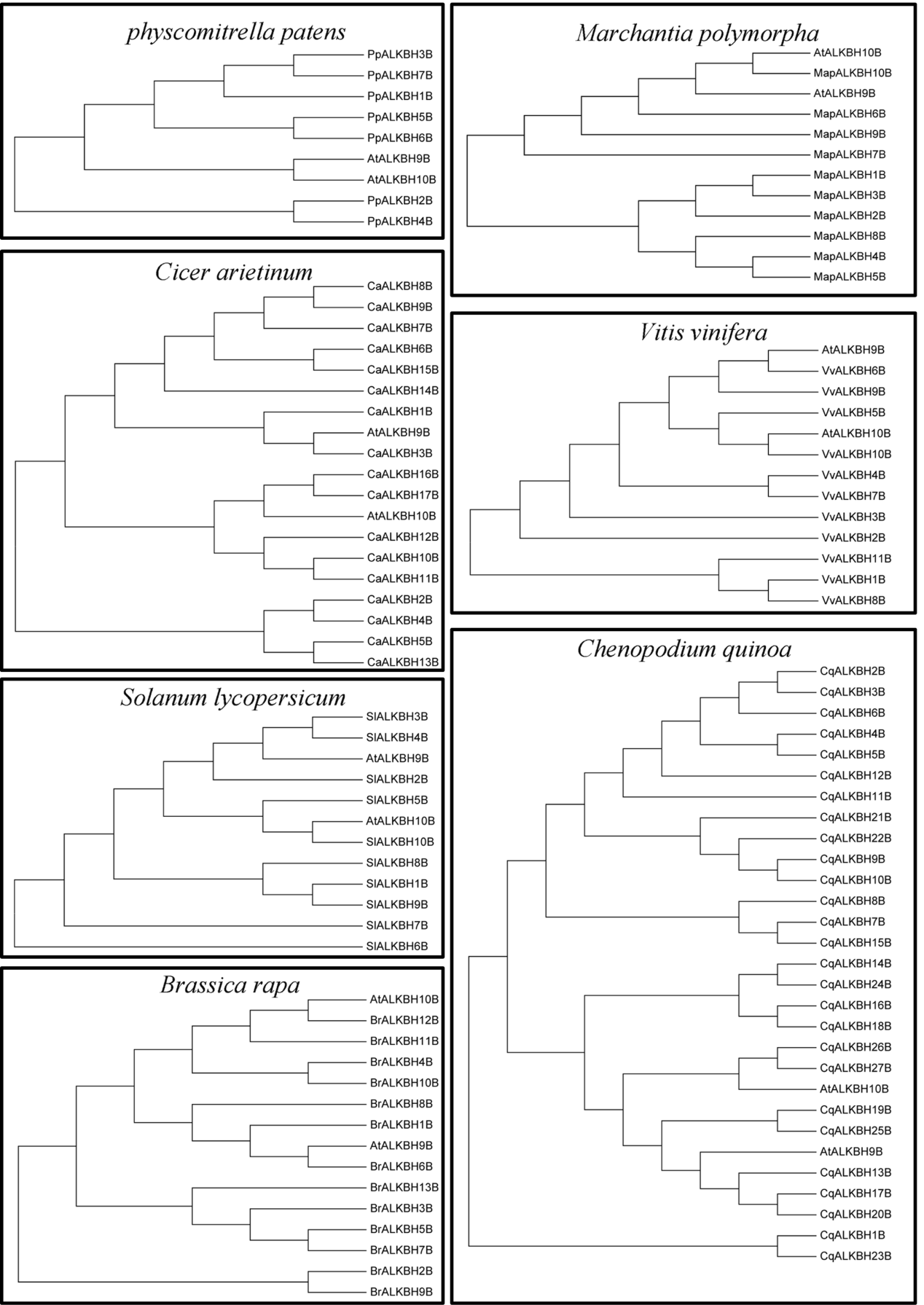
**

Figure S2. Orthologous protein of *Arabidopsis* atALKBH9 and atALKBH10 were identified by phylogenetic analysis among *Physcomitrella patens, Cicer arietinum, Solanum lycopersicum, Vitis vinifera, Marchantia polymorpha, Brassica rapa* and *Chenopodium quinoa*.


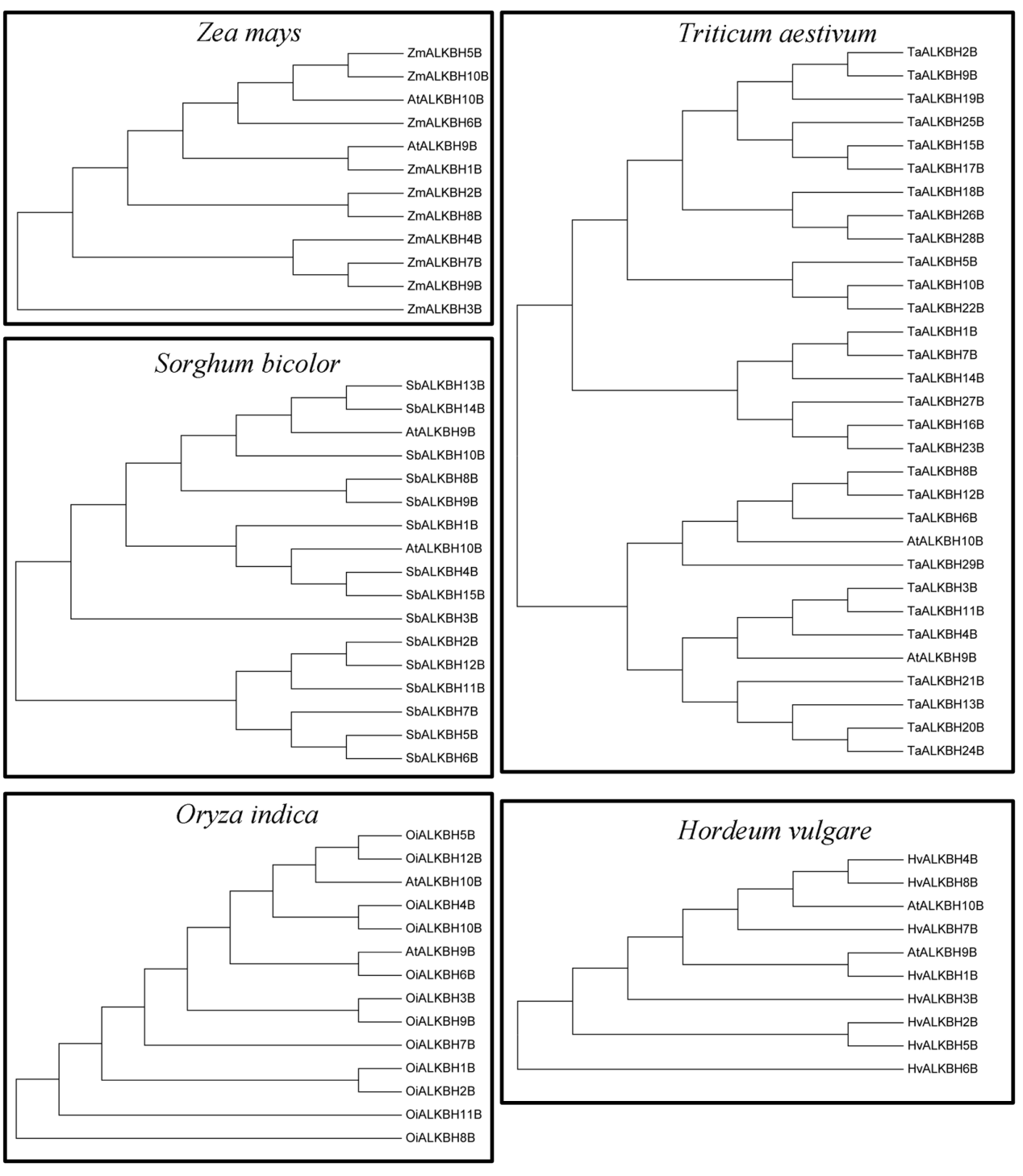


Figure S3. Orthologous protein of *Arabidopsis* atALKBH9 and atALKBH10 were identified by phylogenetic analysis among *Zea mays, Triticum aestivum, Sorghum bicolor, Oryza indica* and *Hordeum vulgare*.


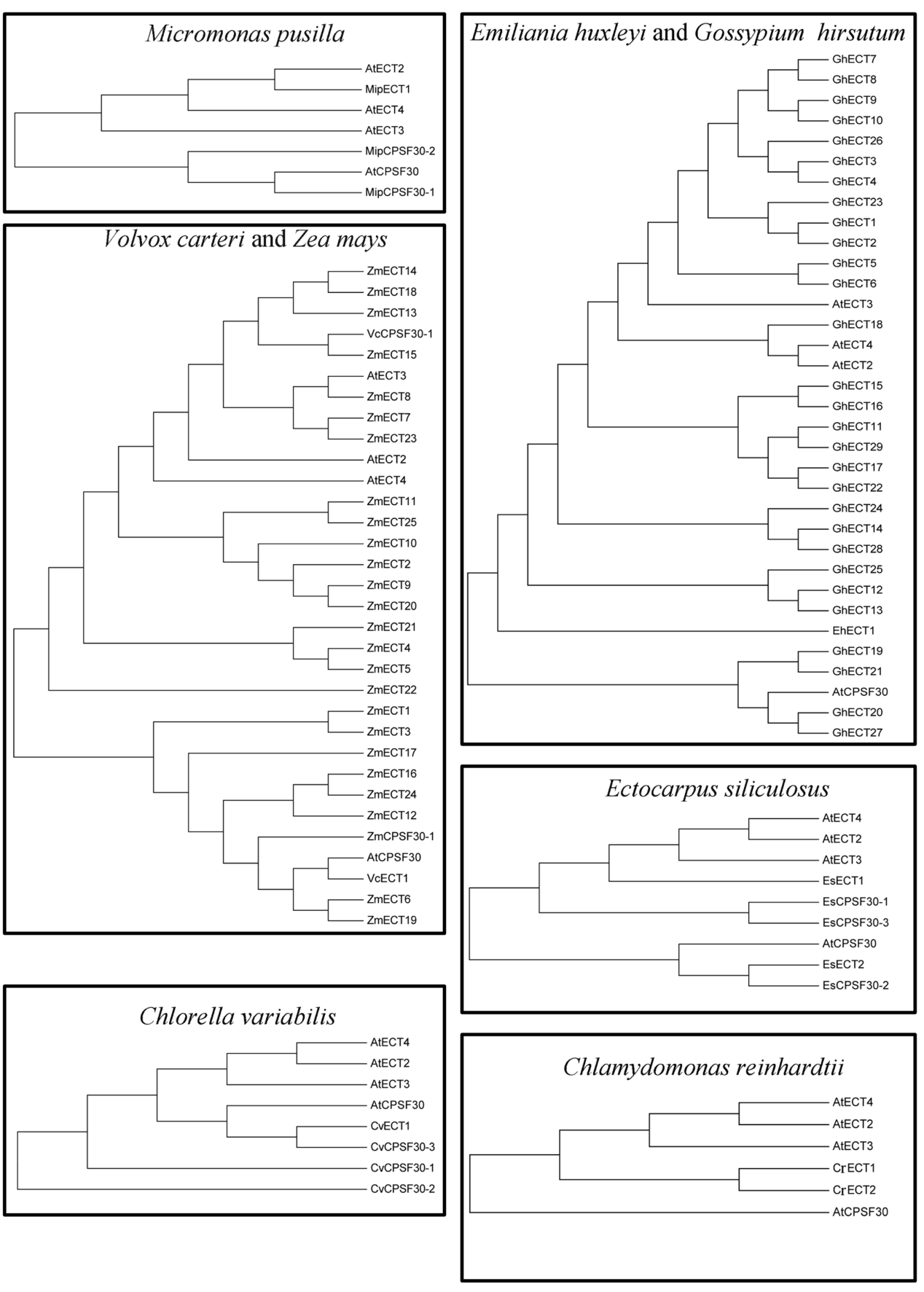


Figure S4. Orthologous protein of *Arabidopsis* ECT2, ECT3 and ECT4 were identified by phylogenetic analysis among *Zea mays, Micromonas pusilla*, *Emiliania huxleyi*, *Volvox carteri*, *Gossypium hirsutum, Ectocarpus siliculosus*, *Chlorella variabilis* and *Chlamydomonas reinhardtii*.


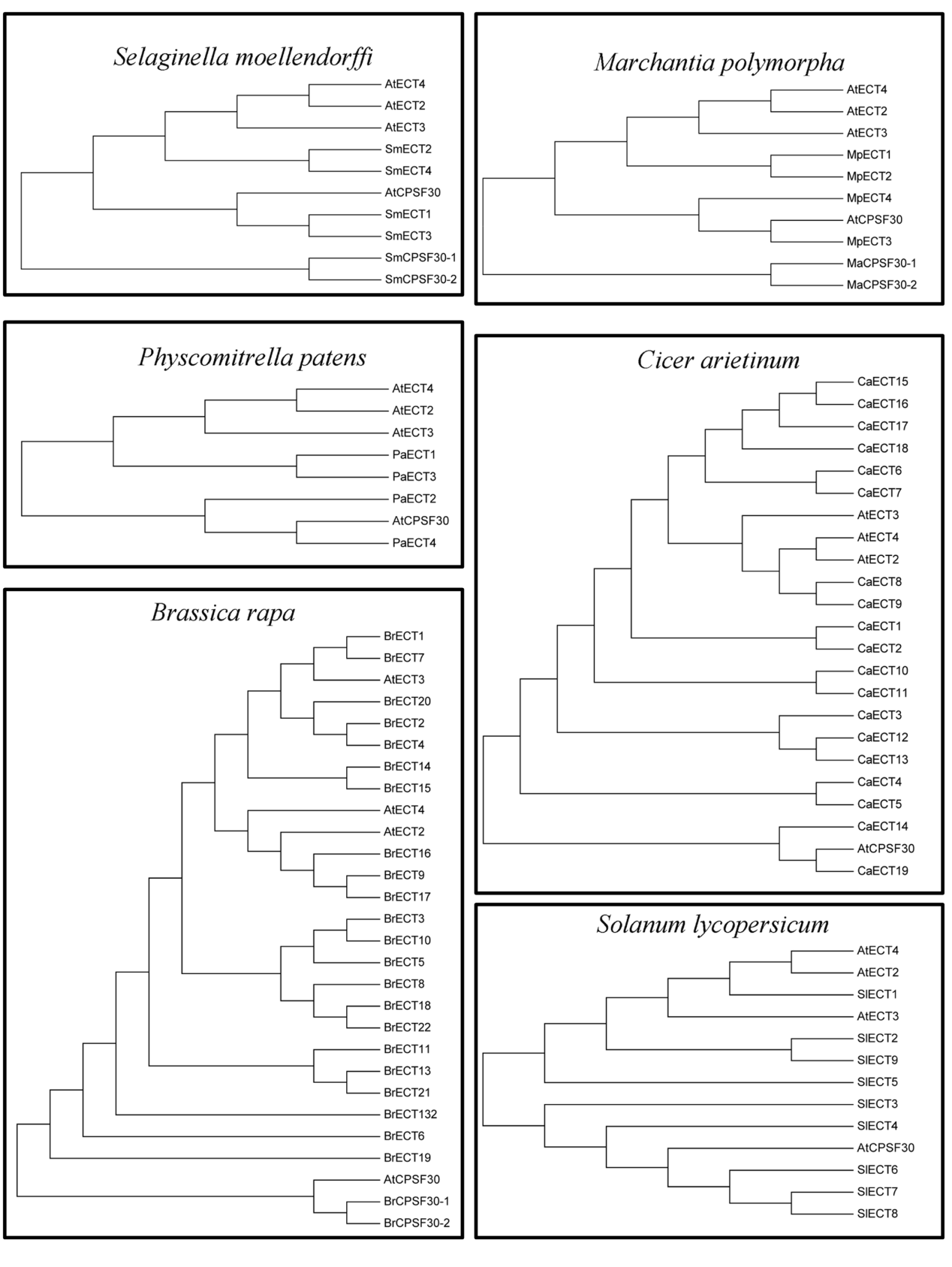


Figure S5. Orthologous protein of *Arabidopsis* ECT2, ECT3 and ECT4 were identified by phylogenetic analysis among *Selaginella moellendorffi*, *Physcomitrella patens, Cicer arietinum, Solanum lycopersicum, Marchantia polymorpha* and *Brassica rapa*.


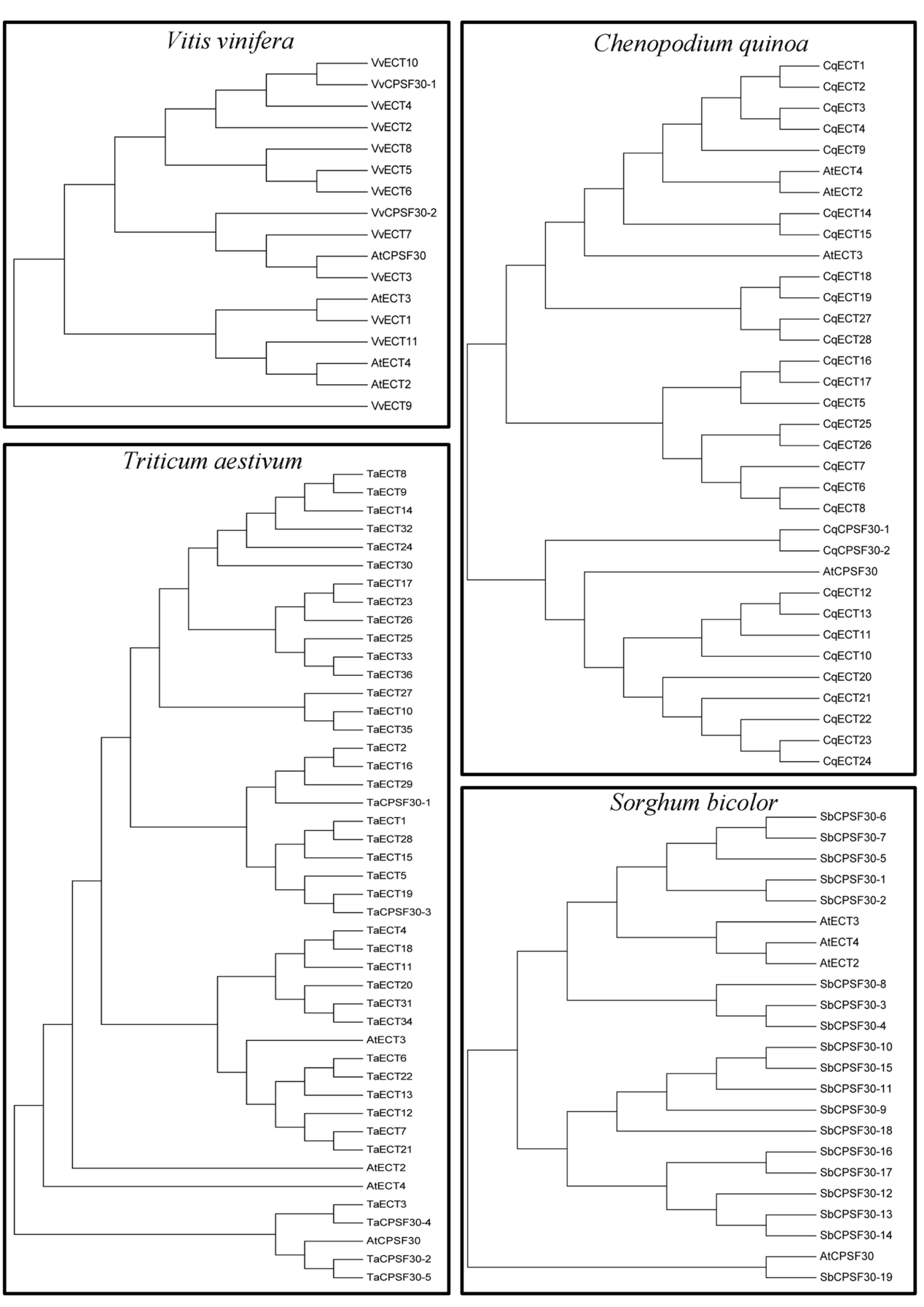


Figure S6. Orthologous protein of *Arabidopsis* ECT2, ECT3 and ECT4 were identified by phylogenetic analysis among *Vitis vinifera, Chenopodium quinoa, Triticum aestivum* and *Sorghum bicolor.*


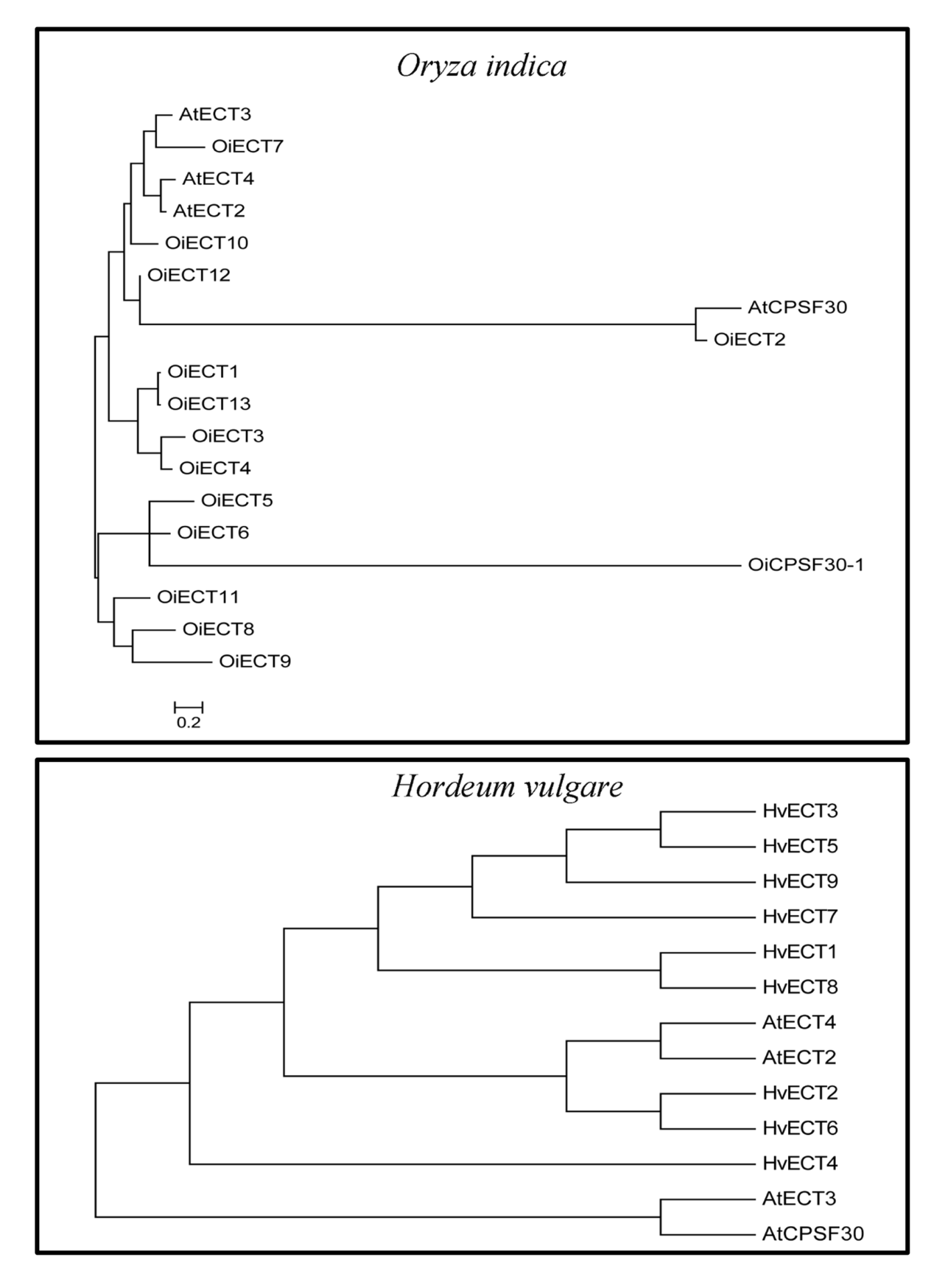


Figure S7. Orthologous protein of *Arabidopsis* ECT2, ECT3 and ECT4 were identified by phylogenetic analysis among *Oryza indica* and *Hordeum vulgare*.
